# Needle-Related Procedural Pain: An Integrated Nervous-System Framework from Tissue Mechanics to Central Processing—A Systematic Review

**DOI:** 10.3390/medsci14060658

**Published:** 2026-10-10

**Authors:** Jorge Ortega-Márquez, Lucas Camargo, Gabriella Freaza, Luísa Melo, Felipe Fregni

**Affiliations:** 1 Office for Graduate Education, Harvard Medical School, Boston, MA 02115, USA; jorge_ortegamarquez@hms.harvard.edu; 2 Neuromodulation Center and Center for Clinical Research Learning, Spaulding Rehabilitation Hospital and Massachusetts General Hospital, Harvard Medical School, Boston, MA 02115, USA; lcamargo@mgh.harvard.edu (L.C.); gabyfreaza@gmail.com (G.F.); lummelo15@gmail.com (L.M.)

**Keywords:** pain modulation, procedural-pain, injection pain, medical devices, biomedical innovation

## Abstract

**Background/Objectives**: Needle procedures cause acute pain through mechanical, chemical, sensory, and affective processes. We synthesized human and animal evidence and developed a framework spanning peripheral nociception, spinal/trigeminal modulation, central processing, and clinical outcomes. **Methods**: This PRISMA 2020 review was registered in PROSPERO (CRD420261477391). PubMed/MEDLINE, Embase, and Web of Science were searched from inception through June 2026. Eligible studies examined pain or mechanistic outcomes during needle procedures. Evidence was classified as peripheral, central, or mixed. Human comparisons were pooled as Hedges’ *g* using random-effects models. **Results**: Thirty-five studies were included: 18 peripheral (51.4%), 13 mixed central peripheral (37.1%), and four central (11.4%). Larger-diameter needles increased pain (painful insertions: 63% with 23-gauge versus 31% with 32-gauge; *p* < 0.001), while bevel geometry, injection pressure, and volume further modulated pain. A 1600 µL volume produced 7.2 mm more pain on a 100 mm scale than smaller volumes (*p* < 0.001). Warming local anesthetic reduced pain from 35.3 to 15.0 mm (*p* < 0.001). Counter stimulation, vibration, cold, distraction, virtual reality, and controlled or automated delivery reduced pain, anxiety, and autonomic responses. Across seven comparisons (625 participants), interventions reduced pain overall (Hedges’ *g*, −0.86; 95% CI, −1.71 to −0.02; *I*^2^ = 77.5%), with differences by nervous-system level (central, −1.65 [−2.60 to −0.70]; peripheral, −1.46 [−4.92 to 1.99]; mixed, −0.36 [−0.99 to 0.28]; subgroup *p* = 0.018). Most studies had moderate risk of bias (62.5%). **Conclusions**: Needle-related pain is a multilevel process. Mechanism-informed interventions reduce pain, although effects vary by mechanism and context. Future studies should integrate peripheral, behavioral, and central measures and adopt standardized outcomes to identify modifiable targets and support mechanism-informed, patient-centered pain reduction.

## 1. Introduction

Pain is not a passive readout of tissue injury but a multidimensional sensory and emotional experience shaped by biological, psychological, and contextual factors [1,2]. Accordingly, mechanistic pain research must explain not only how nociceptive signals are generated and amplified, but also how they are inhibited and how relief emerges. Pain reduction is an active neurobiological process involving peripheral modulation, spinal and trigeminal gating, descending control, expectation, attention, and reward-related circuitry [3,4,5]. This distinction is clinically important because even brief acute pain can provoke distress, reinforce avoidance, and alter subsequent health-care behavior. Painful procedures—including surgery, wound care, venipuncture, intravenous cannulation, vaccination, injections, lumbar puncture, and tissue biopsy—are among the most frequent iatrogenic sources of acute pain. Although often considered transient or unavoidable, inadequately managed procedural pain may compromise cooperation, reduce satisfaction, intensify anxiety, and discourage engagement with future care [6,7,8]. These consequences make it essential to understand how procedural pain is generated and how it can be reduced.

Procedural pain arises through interacting processes that unfold before, during, and after tissue stimulation [2,3]. Mechanical deformation and tissue injury activate peripheral nociceptors [2,9]. Injectate acidity, temperature, viscosity, and inflammatory mediators may recruit or sensitize chemosensitive channels and receptors [10], while fluid delivery can increase interstitial pressure and tissue distension [10,11,12]. These ascending signals are filtered within spinal or trigeminal circuits and transformed by supraspinal processes, including salience, threat appraisal, prior experience, attention, expectation, and descending inhibition [3,4,13]. The same framework explains why relief can be produced through mechanistically distinct routes. These include reducing the initiating stimulus, limiting tissue deformation or chemical irritation, engaging competing non-nociceptive input, attenuating anticipatory threat, and redirecting attention [4,7,13,14]. Yet these processes are commonly studied in isolation, and intervention reviews have generally emphasized whether a technique reduces reported pain rather than how that reduction is produced [7,14]. Consequently, how the physical characteristics of a procedure relate to peripheral transduction, central pain construction, and pain-reduction mechanisms remains underexplored.

Needle-related procedures are both clinically and experimentally informative for advancing the understanding of procedural pain. At least 16 billion injections are administered worldwide each year, making injections one of the most common health-care procedures [15]. These procedures provide a useful model because pain can be examined at successive stages, from skin or mucosal puncture and needle advancement to fluid delivery, and from peripheral nociceptor activation to central nervous system processing. Pain begins when puncture activates peripheral nociceptors, may continue during needle advancement, and can intensify when fluid delivery causes tissue distension or chemical irritation [9,10,11,12]. Needle diameter influences insertion pain and tissue trauma [9], while needle-tip geometry may affect injection pain and needle deformation [16]. Injection pressure, volume, anatomical site, and formulation properties can further modify the nociceptive input generated during delivery [10,11,12]. Once generated, this input is modulated within spinal or trigeminal circuits before engaging cortical networks involved in sensory, affective, and attentional processing [3,4]. Needle puncture evokes measurable cortical activity in infants, while observation of needle-related stimuli recruits cortical regions involved in pain and emotional processing in adults [17,18]. Together, these findings suggest that both nociceptive input and the perceived threat associated with needles contribute to needle-related pain. Repeated coupling of needles with pain or distress may, in turn, reinforce needle fear [6,8]. This fear is common across the lifespan and can contribute to delayed or avoided care [6]. The interaction among nociceptive input, central modulation, and learned threat provides a rationale for physical and psychological pain-reduction approaches, including modifications to needle design and delivery, cooling, pressure, vibration, and distraction [7,10,14,16,19]. Despite this evidence, the field lacks an integrated nervous-system framework explaining how needle-related pain is generated. Such a framework could not only advance mechanistic understanding and guide pain-modulation strategies but also inform the development of novel delivery technologies—including subcutaneous injection devices and microneedle platforms—that minimize tissue injury and perceived threat while preserving therapeutic effectiveness.

This systematic review aimed to synthesize evidence from human and animal studies on the mechanisms that generate and reduce needle-related pain. We examined how peripheral nociceptive input interacts with spinal, trigeminal, and central modulation, and how these processes are influenced by procedural and device-related factors. We hypothesized that needle-related pain is best explained by an integrated, multilevel model rather than a single isolated pathway. Defining this model may identify modifiable mechanisms and support the development of more targeted, effective, and patient-centered pain-reduction strategies.

## 2. Materials and Methods

This systematic review and meta-analysis was conducted in accordance with the Preferred Reporting Items for Systematic Reviews and Meta-Analyses (PRISMA) 2020 statement and the Cochrane Handbook for Systematic Reviews of Interventions [20,21]. The protocol for this study was registered in the International Prospective Register of Systematic Reviews (PROSPERO) under the registration number CRD420261477391. PubMed/MEDLINE, Embase, and Web of Science were searched from database inception through June 2026. Search terms combined concepts related to needle- or injection-related pain with candidate mechanisms, including nociception, tissue injury or distension, inflammation, injection pressure, flow rate, volume, controlled delivery, vibration, gate control, counterstimulation, and sensory modulation. Complete database search strategies are provided in Supplementary Table S1.

### 2.1. Study Selection

We included human and animal studies investigating pain intensity or neurophysiological, inflammatory, biomechanical, chemical, psychological, sensory-modulatory, or central mechanisms associated with needle insertion, injection, venipuncture, vaccination, acupuncture, biopsy, or local-anesthetic delivery. Human and animal studies were eligible when they directly examined needle-tissue interactions or another process with a clear mechanistic link to pain. Experimental, observational, and mechanistic designs were eligible; reviews, editorials, letters, protocols, conference abstracts, and case reports were excluded. After deduplication, titles and abstracts were screened, followed by full-text assessment of potentially eligible reports. Three reviewers (G.F., L.M., L.C.) independently conducted study selection and data extraction, and disagreements were resolved by a third reviewer (J.O.). Extracted variables included author, publication year, study design, population or model characteristics, needle-related procedure, intervention or exposure, comparator, pain measure and assessment time point, mechanistic outcomes, and numerical data required for synthesis.

### 2.2. Risk-of-Bias Assessment

Two reviewers (G.F. and L.M.) independently assessed methodological quality, with disagreements resolved by a third reviewer (L.C.). Human studies were evaluated using design-specific Joanna Briggs Institute checklists: the 13-item checklist for randomized controlled trials, the nine-item checklist for quasi-experimental studies, and the eight-item checklist for analytical cross-sectional studies. Animal experiments were assessed using the 10-domain SYRCLE risk-of-bias tool [22,23]. Items were rated as yes, no, unclear, or not applicable. For descriptive summary, the proportion of applicable items rated yes was categorized as low risk (at least 80%), moderate risk (50–79%), or high risk (less than 50%). Because these instruments do not prescribe universal numerical cutoffs, these review-defined categories were interpreted alongside domain-level judgments.

### 2.3. Data Synthesis

Studies were classified by the principal nervous-system level implicated in the proposed mechanism: peripheral, spinal/trigeminal, central, or mixed. They were also grouped by dominant mechanistic theme: mechanical puncture and needle design; injection pressure, volume, and tissue distension; chemical, formulation, or receptor mechanisms; psychological and sensory modulation; central cortical, affective, or attentional mechanisms; and tissue-depth or referred-pain mechanisms. Classification was based on the procedure, biological rationale, measured outcomes, and authors’ interpretation. All included studies were synthesized narratively by nervous-system level and mechanistic theme, with human, animal, and model-based evidence distinguished.

### 2.4. Statistical Analysis

Statistical analyses were conducted in in R version 4.6.1 (R Foundation for Statistical Computing, Vienna, Austria) using the meta package (version 8.2-1) [24]. Continuous data were extracted as means and standard deviations (SDs). When only the standard error of the mean was reported, it was converted to an SD using SD = SEM × *√n*. Means and SDs were derived from reported category counts when this could be done exactly; uncertain graph-estimated or assumption-based values were not used in the primary meta-analysis.

Meta-analyses were restricted to independent, parallel-group human comparisons reporting a direct pain outcome and providing, or permitting reliable derivation of, the sample size, mean, and SD for each group. Reported pain was selected for quantitative analysis as the common clinical outcome through which interventions with different proposed mechanisms could be compared. Paired, crossover, and repeated-measures comparisons were not pooled when a paired effect estimate or within-participant correlation was unavailable. Animal and model-based studies, non-pain outcomes, and duplicate outcomes from the same sample were excluded from the primary pool. For multi-arm studies or studies with a shared comparator, one comparison was selected to avoid double counting. Because pain was measured using different scales, effects were expressed as Hedges’ g, the small-sample-corrected standardized mean difference, with 95% confidence intervals; negative values indicated less pain with the intervention. Effects were pooled using an inverse-variance random-effects model. Between-study variance (tau-squared) was estimated by restricted maximum likelihood, and pooled confidence intervals were calculated using the Hartung–Knapp method. Statistical heterogeneity was assessed using Cochran’s Q, I-squared, and tau-squared. An exploratory subgroup analysis evaluated differences among peripheral, central, and mixed nervous-system classifications. Subgroup estimates were pooled only when at least two independent comparisons were available. Funnel-plot asymmetry and regression tests for small-study effects were not performed because fewer than 10 independent comparisons were available [25].

## 3. Results

The database search identified 5865 records. After removing 2091 duplicates, 3774 records were screened by title and abstract, of which 3646 were excluded. The full texts of 128 reports were assessed for eligibility, and 93 were excluded, leaving 35 studies included in the review; see Figure 1. Across the 35 included studies, needle-related pain was investigated through six complementary mechanistic domains (Figure 2). Based on primary nervous-system classification, peripheral mechanisms were most common (*n* = 18, 51.4%) [9,11,12,16,26,27,28,29,30,31,32,33,34,35,36,37,38,39], followed by mixed CNS–PNS mechanisms (*n* = 13, 37.1%) [40,41,42,43,44,45,46,47,48,49,50,51,52] and central mechanisms (*n* = 4, 11.4) [17,18,53,54]. Across mechanism categories, psychological and sensory-modulatory mechanisms were the most frequent (*n* = 11, 31.4%) [40,41,42,43,44,45,46,47,48,49,50], followed by chemical/formulation/receptor mechanisms (*n* = 7, 20%) [26,28,29,33,35,36,37], mechanical puncture/needle design (*n* = 5, 14.3%) [9,16,27,32,34], injection pressure/volume/tissue distension (*n* = 6, 17.1%) [11,12,30,31,38], central cortical/affective/attention mechanisms (*n* = 4, 11.4%) [17,18,53,54], and tissue-depth/referred-pain mechanisms (*n* = 2, 5.7%) [51,52]. Details of the 35 studies included are displayed in Supplementary Tables S1–S5.

### 3.1. Integration of Mechanistic Evidence Across the Nociceptive Pathway

Mapping the included evidence onto the ascending nociceptive pathway revealed four interconnected anatomical and functional levels (Figure 3). Fourteen studies primarily informed peripheral tissue transduction and nociceptor activation, while two examined dorsal-root-ganglion or spinal encoding. Twelve studies investigated mixed sensory–attentional mechanisms, including counterstimulation, vibration, distraction, and device-related modulation. Six studies provided evidence related to central representation or brain-level modulation. Collectively, the evidence supports a sequential but bidirectionally modulated framework. Needle–tissue interactions and injectate properties initiate peripheral nociceptive input, which is encoded and modulated within ganglionic and spinal circuits before engaging sensory, attentional, affective, and cortical networks. Modulation may occur at each level rather than through a single pain-reduction pathway.

### 3.2. Mechanical Pain During Needle Insertion

The first phase of needle-induced pain occurs at the needle–tissue interface, before injectate delivery. Five studies supported this peripheral mechanical pathway in which penetration of the skin and underlying tissues produces local deformation, compression, and disruption of tissue structures. These mechanical forces activate cutaneous and subcutaneous nociceptors, particularly Aδ and C fibers, generating the immediate sharp or pricking sensation associated with needle insertion. Edlich et al. (1987) [32] supported the role of needle gauge and syringe mechanics, reporting that 30G needles caused less puncture pain than larger needles on an ordinal pain scale and recommending slow infiltration over approximately 10 s. Also, Arendt-Nielsen et al. (2006) [9] showed that larger needle diameter increased painful insertion frequency, with painful insertions occurring in 63% of 23G insertions, 54% of 27G insertions, and 31% of 32G insertions (*p* < 0.001), while bleeding insertions were approximately 1.3 times more painful than non-bleeding insertions (*p* = 0.004), supporting a relationship between tissue trauma and pain. Additionally, Dau et al. (2017) [16] found that scalpel-bevel needles produced lower pain scores than triple-bevel and regular-bevel needles (2.1 ± 1.2 vs. 3.5 ± 1.6 and 3.4 ± 1.0, respectively; *p* < 0.001). Besides, Moaddabi et al. (2024) [34] showed that procedural mechanics also modified pain, with mean pain scores decreasing from 7.44 ± 0.88 using a conventional syringe without optimized angle to 0.67 ± 0.50 using computer-controlled local anesthetic delivery (CCLAD) with a different needle angle (*p* < 0.001). In a non-human study, Alghurair et al. (2013) [27] showed in an artificial-skin model that plasma-treated needles reduced insertion force by approximately half compared with untreated needles, suggesting that cutting-edge roughness and sharpness influence puncture mechanics. The needle and procedural characteristics evaluated across these studies, including needle diameter, bevel geometry, insertion angle and delivery systems, and needle-surface properties are summarized in Figure 4.

### 3.3. Injection Pressure, Volume, and Tissue Distension

After needle insertion, fluid delivery can amplify pain by increasing local interstitial pressure and producing tissue distension, which mechanically activates nociceptive Aδ and C fibers. Kudo et al. (2005) [11] directly measured pressure during oral mucosal anesthesia and found that injection pressure correlated with both pain (rs = 0.579, *p* = 0.001) and state anxiety (rs = 0.479, *p* = 0.010), recommending that pressure remain below 306 mmHg. In subcutaneous injections, Heise et al. (2014) [12] found no effect of injection speed (*p* = 0.833), but 1600 µL produced 7.2 mm more pain on a 100 mm VAS than either 400 or 800 µL (both *p* < 0.001). Similarly, Zijlstra et al. (2018) [38] found that pain scores were generally low, usually <20 mm on a 100 mm VAS, but speed did not meaningfully increase pain. In contrast Berteau et al. (2015) [30] found that viscosity affected pain (*p* < 0.001), but volume (*p* = 0.89) and flow rate (*p* = 0.79) were not significant within the tested ranges. In a non-human study, Deng et al. (2026) [31] extended this pathway using a computational hydrogel model, showing that post-injection tissue stress peaked at approximately 60–100 min, reached approximately 10.8 kPa, exceeded the reported nociceptor activation threshold of 6–9 kPa, and approached equilibrium after approximately 400 min. In a formalin-induced pain model in rats, Jin et al. (2025) [39] found that automated injection reduced paw licking by 66.0% during the first 15 min (*p* < 0.05) and attenuated spinal nociceptive and neuroinflammatory markers, including c-fos, GFAP, Oprl1, Htr2a, and Oprm1. Although pressure and tissue distension were not directly measured, these findings provide indirect evidence that greater control of injection delivery may reduce immediate nocifensive behavior and downstream spinal nociceptive activation. Taken together, these studies suggest that injection pain may be influenced by the interaction among delivery pressure, volume, flow characteristics, and tissue compliance.

### 3.4. Chemical and Receptor-Mediated Nociception

A second peripheral pathway involved chemical or receptor-mediated activation of nociceptors. In the included studies, this pathway was driven by changes in proton concentration, inflammatory mediators, drug formulation, temperature, and ligand-specific receptor signaling. Low extracellular pH can directly excite peripheral nociceptors through acid-sensing ion channels and TRPV1. Rukwied et al. (2007) [36] showed that low-pH solutions produced rapid pain within approximately 5 s, declining over 15–20 s. When prostaglandin E2 was combined with low pH, pain increased during a later phase between 20 and 100 s in both skin and muscle, consistent with inflammatory sensitization of proton-responsive nociceptors, particularly TRPV1, rather than enhancement of the initial proton-evoked response. Meanwhile, Ohmizo et al. (2005) [35] found that propofol formulated with long-chain/medium-chain triglycerides produced less injection pain than long-chain triglyceride propofol; pain score 0 was reported in 63.3% of the long-chain triglyceride/medium-chain triglyceride (LCT/MCT) group compared with 38.2% of the LCT group (*p* < 0.01).

Moreover, temperature changes with warming local anesthetic (42 °C), demonstrated by Aravena et al. (2018) [29], reduced dental injection pain from 35.3 ± 16.71 mm to 15.0 ± 14.67 mm on a 100 mm VAS (*p* < 0.001). In pre-clinical studies, Ami et al. (2015) [28] found that EMG nociceptive reflex responses increased with injection volume and acidity, but not with osmotic pressure up to an osmotic ratio of approximately 5. Besides, Khodorova et al. (2010) [33] showed that endothelin-1 injection pain tachyphylaxis was peripheral and ETA-receptor dependent, with second flinching responses reaching 20 ± 5%, 57 ± 18%, 79 ± 35%, and 100 ± 17% of the first response at 24, 30, 48, and 72 h, respectively. Interestingly, Alfaraj et al. (2022) [26] observed that caffeine potentiated the local anesthetic effect of lidocaine, reduced pressure sensation compared with control, enhanced thermal pain-relieving effects, and modulated dorsal root ganglia expression of Nav1.3, Nav1.7, and Nav1.8. While Udagawa et al. (2018) [37] showed that chemical nociception also varied by tissue depth: subcutaneous glutamate induced more pain and unpleasantness than intramuscular glutamate, while bone-surface glutamate increased pressure pain sensitivity more than intramuscular glutamate. Together, these studies indicate that injectate chemistry, pH, inflammatory mediators, temperature, glutamate, endothelin-1, and receptor/ion-channel pathways contribute to injection-related pain.

### 3.5. Tissue Depth and Referred-Pain Mechanisms

Pain arising from deeper tissues may differ from cutaneous pain because muscle nociceptors generate more diffuse and poorly localized afferent input, while convergence of muscle and cutaneous afferents onto shared spinal and supraspinal neurons may cause pain to be perceived beyond the stimulated site. The extent of referred pain may also be influenced by peripheral sensitization and the efficiency of descending inhibitory control. Uematsu et al. (2011) [52] showed that muscle and cutaneous pain recruited partially distinct central networks. Nociceptive muscle stimuli activated the anterior midcingulate cortex, anterior and posterior insula, thalamus, pre-supplementary motor area, dorsolateral prefrontal cortex, and inferior parietal lobule, whereas secondary somatosensory cortex responses were more prominent for cutaneous pain. While Sago et al. (2023) [51] found that referred sensations were associated with increased mechanical sensitivity and reduced conditioned pain modulation (*p* < 0.05), and that referred sensation incidence, frequency, intensity, and area decreased during painful conditioning and after local anesthetic block (*p* < 0.05). These findings support a mixed CNS–PNS pathway involving tissue depth, afferent convergence, referred sensation, and descending modulation.

### 3.6. Cortical, Emotional, and Sensory Modulation of Pain

Six clinical studies showed that non-nociceptive sensory input can reduce injection pain through spinal/trigeminal gating, sensory modulation, and attentional mechanisms. Pressure, vibration, and cold may activate mechanoreceptive fibers that inhibit nociceptive transmission at spinal or trigeminal sensory relays, while distraction, virtual reality, and syringe camouflage redirect attention and reduce anticipatory anxiety. Aminabadi et al. (2008) [40] found that Sound–Eye–Motor pain reaction scores were the highest with simple anesthesia (8.25), lower with counterstimulation (5.07), and the lowest with counterstimulation plus distraction (3.41), with significant group differences (*p* < 0.05). Years before, Barnhill et al. (1996) [42] had tested manual pressure before intramuscular immune globulin injection, supporting tactile counterstimulation as a possible gate-control mechanism, although exact pain values were not reliably extracted. Moreover, Hegde et al. (2019) [47] reported lower pulse rate, “Face, Legs, Activity, Cry, Consolability” (FLACC) scores, and Wong–Baker pain scores with a vibration/distraction device compared with conventional injection. Recently, Jabeen et al. (2025) [48] found that a mucosal vibrator significantly reduced subjective and objective pain scores compared with benzocaine gel (*p* < 0.05), although pulse rate did not significantly change.

Also, Hambouta et al. (2025) [46] found that Buzzy (MMJ Labs, LLC, Atlanta, GA, USA) cold/vibration did not significantly differ from topical gel for buccal anesthesia but significantly reduced subjective and objective pain during palatal and intrapapillary anesthesia comparisons. Using a different approach, Hagwane et al. (2025) [45] found that camouflaged syringes produced lower Wong–Baker Faces Pain Rating Scale scores than insulin syringes before treatment (5.83 ± 0.76 vs. 6.45 ± 1.24; *p* = 0.001) and after treatment (3.58 ± 0.74 vs. 4.17 ± 1.37; *p* = 0.005), with additional benefit from thumb-pressure counterstimulation; however, behavioral FLACC findings should be interpreted cautiously because the extracted table notes inconsistency between table values and the authors’ narrative conclusion. Additionally, Nunna et al. (2019) [50] found no significant difference in pain between counterstimulation and virtual reality, with WBFPS 2.97 ± 2.491 vs. 3.03 ± 2.022 (*p* = 0.770) and VAS 3.06 ± 2.313 vs. 2.94 ± 1.765 (*p* = 0.991). However, pulse rate during injection was lower with VR than counterstimulation (93.34 ± 14.353 vs. 101.67 ± 17.346; *p* = 0.032) and remained lower after injection (91.56 ± 12.679 vs. 100.51 ± 15.399; *p* = 0.010), while Venham anxiety scores improved significantly within the VR group (*p* = 0.008). These studies suggest that sensory modulation can influence pain, anxiety, and autonomic response, but the relative contribution of peripheral gating versus attention varies across interventions.

Cortical mechanisms were supported by studies of attention, distraction, cortical nociceptive processing, visual threat, and device-related anxiety. Emotional state, expectation, and prior needle experiences can shape pain by modifying attention and threat appraisal before and during injection. Anticipatory anxiety and negative expectations increase vigilance toward the needle and amplify activity in pain- and emotion-related cortical networks, whereas distraction and reassurance can reduce the salience of nociceptive input. Fowler-Kerry et al. (1987) [53] studied 200 children receiving immunizations and found that pain scores were lower with distraction-based conditions: control 1.78, suggestion 1.59, distraction 1.34, and distraction plus suggestion 1.07 (*p* = 0.007), whereas suggestion alone was less effective than distraction. Also, Padmanabh et al. (2025) [54] found that audiovisual/VR distraction reduced pain/anxiety scores more than control: in one intervention group, VAS decreased from 6.58 ± 0.996 to 4.67 ± 0.779 (*p* = 0.001), and in another from 6.50 ± 1.567 to 4.42 ± 0.669 (*p* < 0.001), while the control group showed a smaller non-significant reduction from 6.42 ± 1.379 to 6.00 ± 1.128 (*p* = 0.209). In an EEG study, Verriotis et al. (2015) [17] reported that routine inoculation needle prick evoked distinct EEG event-related vertex potentials in infants, with greater negative–positive component amplitudes at 12 months than at 1–2 months, indicating developmental changes in cortical nociceptive processing. Ushida et al. (2008) [18] observed that virtual needle stimuli activated pain- and emotion-related brain networks, particularly in participants preconditioned with real needle puncture; the preconditioned group showed greater activation in the anterior insula, inferior frontal gyrus, precentral gyrus, and cerebellum.

In pediatric studies, Badr et al. (2026) [41] randomized 112 children to manual syringe, computer-controlled local anesthetic delivery, vibration-assisted injection, or needle-free jet injection; the manual syringe group showed higher Wong–Baker pain, FLACC distress, and Venham anxiety during anesthesia than alternative techniques (*p* ≤ 0.05), while CCLAD showed the most consistent benefit. Karuppiah et al. (2026) [49] found that pain/anxiety scores were lower with Star Pen (Guilin Woodpecker Medical Instrument Co., Ltd., Guilin, Guangxi, China) and insulin syringe than with conventional syringe; Animated Emoji Scale scores increased from 1.27 ± 0.458 to 2.67 ± 0.816 with Star Pen, 1.13 ± 0.352 to 2.73 ± 0.799 with insulin syringe, and 1.33 ± 0.617 to 3.00 ± 0.655 with conventional syringe. Pulse rate differed significantly before (*p* = 0.004) and during injection (*p* = 0.002). Together, these studies show that needle-related pain is transformed by central threat processing, attention, prior experience, device perception, anxiety, and behavioral response.

### 3.7. Administration-Related Findings for Accuracy and Reliability

Administration-related findings emerged across peripheral and cortical mechanism categories rather than constituting a single isolated pathway. In this context, administration accuracy refers to whether the intended dose is delivered to the intended anatomical site and tissue depth, whereas administration reliability refers to whether the procedure is performed consistently across repeated injections, operators, devices, and clinical settings [56,57]. Most included studies evaluated selected administration parameters associated with pain rather than directly measuring these broader outcomes. Injection pressure and volume were correlated with both pain than injection speed [11,12,38]. Interestingly, Jin et al. (2025) [39] compared a digital automatic syringe (i-JECT™; MediHub Co., Ltd., Gunpo-si, Gyeonggi-do, Republic of Korea) with a conventional manual syringe in a formalin-induced pain model in rats (*n* = 15; five per group). During the first 15 min after injection, the automatic syringe reduced paw-licking episodes by 66.0% compared with the manual syringe (3.4 vs. 10.0; *p* < 0.05). These findings indicate that controlled automated delivery reduced immediate nocifensive behavior and attenuated spinal neuronal and glial responses in this animal model.

Device-based studies showed that altering the administration system can influence pain and anxiety, but effects were not uniform. Flisfisch et al. (2021) [43] found that conventional syringe injection produced approximately threefold higher anxiety-inducement and administration pain than CCLAD, with anxiety-inducement 35.95% vs. 11.85% (*p* < 0.001) and pain during administration 21.3% vs. 7.7% (*p* = 0.005). In contrast, Goodell et al. (2000) [44] found that conventional atraumatic syringe injection produced lower post-injection anxiety, lower pain, and better overall experience than the Wand controlled-rate system (Milestone Scientific, Inc., Livingston, NJ, USA). Moaddabi et al. (2024) [34] showed that combining optimized needle angle with CCLAD produced the lowest pain scores, suggesting that controlled delivery may be most effective when paired with optimized procedural technique. These findings indicate that controlled delivery alone is not sufficient; device design, visual threat, auditory cues, operator technique, tissue site, and patient expectations likely modify the administration experience.

In the context of the pediatric population, studies also showed that administration experience is shaped by device appearance, sensory context, and behavioral response. Camouflaged syringes technique, lower pain/anxiety with less threatening syringe designs and other distraction or vibration techniques shower reduced in pain levels compared to manual syringe delivery [41,45,49]. However, most included studies did not directly evaluate administration reliability. Outcomes such as dose completion, premature removal, handling errors, injection-duration consistency, correct workflow completion, injection-history documentation, or concordance between device-recorded data and patient self-report were rarely measured. Thus, while the literature supports links between injection mechanics, device experience, and patient response, objective administration-integrity outcomes remain underdeveloped.

### 3.8. Effects of Pain-Reduction Interventions on Needle-Related Pain

Seven independent comparisons involving 625 participants (312 intervention and 313 comparator) were included in the meta-analysis. In the random-effects model, interventions reduced needle-related pain overall (Hedges’ g = −0.86, 95% CI −1.71 to −0.02), with substantial heterogeneity (I^2^ = 77.5%). When organized by nervous-system classification, the pooled effect estimates were −1.46 (95% CI −4.92 to 1.99; I^2^ = 84.9%) for peripheral interventions and −0.36 (95% CI −0.99 to 0.28; I^2^ = 31.1%) for mixed interventions. The central classification included one comparison, with an effect estimate of −1.65 (95% CI −2.60 to −0.70). Effect estimates differed across nervous-system classifications (χ^2^ = 8.01, df = 2, *p* = 0.018; Figure 5). Given the observed heterogeneity observed across studies this quantitative analysis is considered exploratory.

### 3.9. Gaps in Study Designs and the Evidence Base

Risk of bias was assessed in 32 studies using the Joanna Briggs Institute critical appraisal checklists for human studies and the SYRCLE tool for animal studies. Among the studies for which an overall classification could be assigned, 20 (62.5%) were rated as having a moderate risk of bias, seven (21.9%) as high risk, and five (15.6%) as low risk. The mean proportion of criteria rated “Yes” was 63.7%, with a median of 69.2%. Three bench, computational-simulation, or technical/device studies were not assigned an overall risk-of-bias classification because no available tool was considered appropriate for their specific designs and methodological characteristics.

Among the 28 human studies assessed using JBI tools [22], 20 were classified as moderate risk, five as low risk, and three as high risk. The most consistent strengths were the use of comparable treatment conditions, appropriate outcome-assessment procedures, and adequate statistical analyses. However, concerns were frequently identified regarding allocation concealment, participant and investigator blinding, and blinding or random selection during outcome assessment. These limitations were particularly common when blinding was difficult because of the nature of the intervention. In contrast, all animal studies assessed using SYRCLE were classified as high risk, primarily because of insufficient reporting of randomization, allocation concealment, random housing, and blinding procedures.

Important gaps were also identified in the types of mechanisms investigated. Only four studies (11.8%) primarily examined cortical, affective, or attentional mechanisms, indicating that the evidence base remains weighted toward peripheral, mechanical, chemical, and psychological aspects of needle-related pain. The central nervous system studies used heterogeneous experimental paradigms and outcome measures, limiting direct comparison across studies. Evidence directly linking cortical responses, attentional processes, emotional expectations, and prior needle experiences with subjective pain and clinical outcomes was limited. Longitudinal studies and mechanistic investigations integrating central nervous system measures with peripheral, autonomic, and behavioral responses were also lacking. Overall, the evidence base was characterized by predominantly moderate risk of bias and limited direct investigation of central pain-processing mechanisms, supporting cautious interpretation of the findings and the need for more standardized and mechanistically integrated research.

## 4. Discussion

This systematic review synthesized 35 human, animal, bench, and computational studies and identified a broad but uneven evidence base for the mechanisms of needle-related pain. The available studies indicate that the experience of pain reflects the interaction of sensory gating, central pain processing, attention, anxiety, expectation, prior experience, device features, and administration technique.

Several associations examined here, including those involving needle diameter, injection technique, pressure, formulation, and anxiety, have been reported previously. The contribution of this review is the systematic appraisal and organization of these findings across proposed levels of pain processing, while distinguishing direct human pain outcomes from indirect mechanistic evidence. Across studies, the effects of factors such as injection volume and speed were not uniform, and few studies directly linked central measures to reported pain. This variability highlights the importance of quantifying the relative contribution of each mechanism rather than assuming that mechanically more noxious stimuli necessarily translate into proportionally greater perceived pain. Interestingly, in our meta-analysis, a statistically significant reduction in pain emerged only when studies targeting different mechanisms were pooled together, whereas the separate mechanistic subgroups did not consistently show significant effects. Although this finding should be interpreted cautiously given the small number of studies and the heterogeneity of the interventions assessed, it may suggest that effective pain reduction during injections requires attention to multiple components of the pain pathway rather than focusing on a single mechanism in isolation.

Moreover, other mechanical factors that would intuitively be expected to increase pain during drug delivery showed inconsistent effects across studies. Fluid deposition can distend tissue and increase local mechanical stress, but the contribution of individual parameters such as volume and injection speed was not uniform. Larger injection volumes were associated with greater pain in some studies, whereas others found no significant volume effect [12,30]. Injection speed likewise did not emerge as a consistent determinant of pain. Faster delivery might theoretically increase the rate of local pressure accumulation and tissue deformation; however, no meaningful effect of injection speed on perceived pain [12,30]. Despite the lack of a clear influence of injection speed in these studies, a more conservative interpretation is appropriate. Injection speed could still have a direct mechanical effect on tissue distension and local pressure, particularly when larger volumes are delivered into tissues with greater resistance or lower compliance [58,59]. Because tissue resistance may vary across anatomical locations and between patients, the contribution of injection speed may be context dependent and should be further investigated rather than considered negligible.

Emotional processing is particularly important because cortical mechanisms can modulate incoming peripheral nociceptive signals. Pain-related cortical activity and functional connectivity were more closely associated with unpleasantness than with intensity across multiple regions, including the bilateral insular and frontal opercular cortices and sensorimotor and frontal areas [60]. The affective component of pain reflects its unpleasantness and motivates protective behavioral responses. Emotional states can modify pain through descending pathways connecting the ACC, PFC, and amygdala with the PAG, brainstem, and spinal cord. Emotional and attentional influences may involve partly different mechanisms: negative emotion primarily increases pain unpleasantness through ACC–PFC–PAG circuitry, whereas attention more directly influences perceived intensity through regions including S1 and the insula [61]. This framework is consistent with the studies included in our review. For example, Ushida et al. (2008) [18] found that viewing a virtual needle activated pain- and emotion-related regions, with greater anterior insula, inferior frontal gyrus, precentral gyrus, and cerebellar activation among participants previously conditioned with actual needle puncture, suggesting that prior experience can strengthen the cortical response to needle-related threat. These findings suggest that prior painful experiences can strengthen cortical responses to needle-related cues and potentially amplify the perceived threat of subsequent procedures. However, clinical trials directly examining how central mechanisms modulate injection-related pain remain limited.

Operator expertise may influence needle-related pain through both technical performance and the context in which the procedure is delivered. Needle selection, insertion angle, anatomical site, penetration depth, pressure, volume, and delivery consistency can affect peripheral nociceptive input, whereas communication, needle visibility, distraction, and tactile or cooling strategies may influence attention, anxiety, and expectations [7,14,62]. Variations in experience and technique may therefore expose patients to inconsistent mechanical forces, tissue disruption, and tissue distension. Automated or computer-controlled devices may reduce this variability by standardizing flow rate and injection pressure, potentially producing more consistent administration and reducing pain associated with rapid or high-pressure delivery [63]. Nevertheless, automation cannot eliminate operator influence because correct device positioning, site selection, patient preparation, and communication still depend on the operator. Future studies should directly assess operator experience and between-operator variability and determine whether automated systems provide more consistent pain and safety outcomes than manual techniques. Although the integration of human and animal studies provides a broader view of proposed mechanisms, it also limits direct comparison. Procedures, experimental settings, and outcomes differ, and animal nocifensive behavior or model-based measures cannot be equated with human-reported pain. Non-human findings therefore provide mechanistic context but do not establish the magnitude of pain or the effectiveness of pain-reduction interventions in patients.

## 5. Conclusions

This review provides a broad synthesis of needle-related pain by integrating mechanical, chemical, sensory, psychological, central, and operator-dependent mechanisms across diverse procedures and populations. This multilevel approach extends previous reviews that focused primarily on specific procedures or pain-reduction interventions. However, the evidence was heterogeneous in study design, population, injection technique, anatomical site, and outcome measurement, limiting direct comparisons. Therefore, these findings should be interpreted as a mechanistic framework that highlights emerging patterns and key knowledge gaps in the field.

Needle-related pain is a multidimensional acute experience shaped by interacting mechanical, chemical, sensory, psychological, and operator-dependent factors. Effective pain reduction will therefore likely require an integrated approach that optimizes needle–tissue interaction, injectate delivery, sensory modulation, and the emotional context of care. Larger, well-designed studies using standardized patient-reported, behavioral, physiological, neural, and administration-related outcomes are needed to identify the most effective strategies for different procedures and populations. Collaboration among patients, clinicians, engineers, and pain scientists will be essential to translate this mechanistic understanding into safer, more consistent, and less distressing injection practices and technologies.

## Figures and Tables

**Figure 1:**
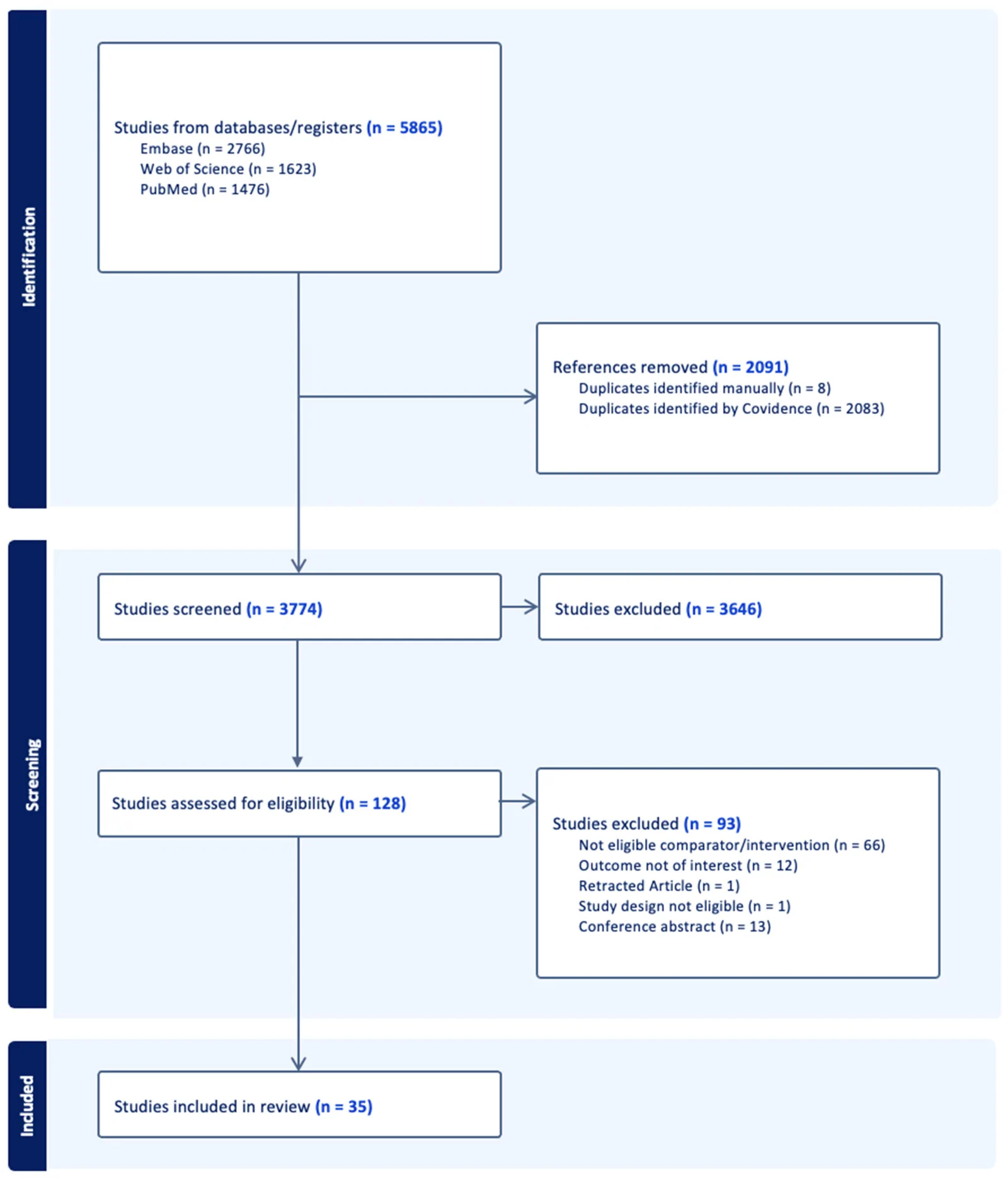
PRISMA flow diagram of study identification, screening, eligibility assessment, and inclusion.

**Figure 2:**
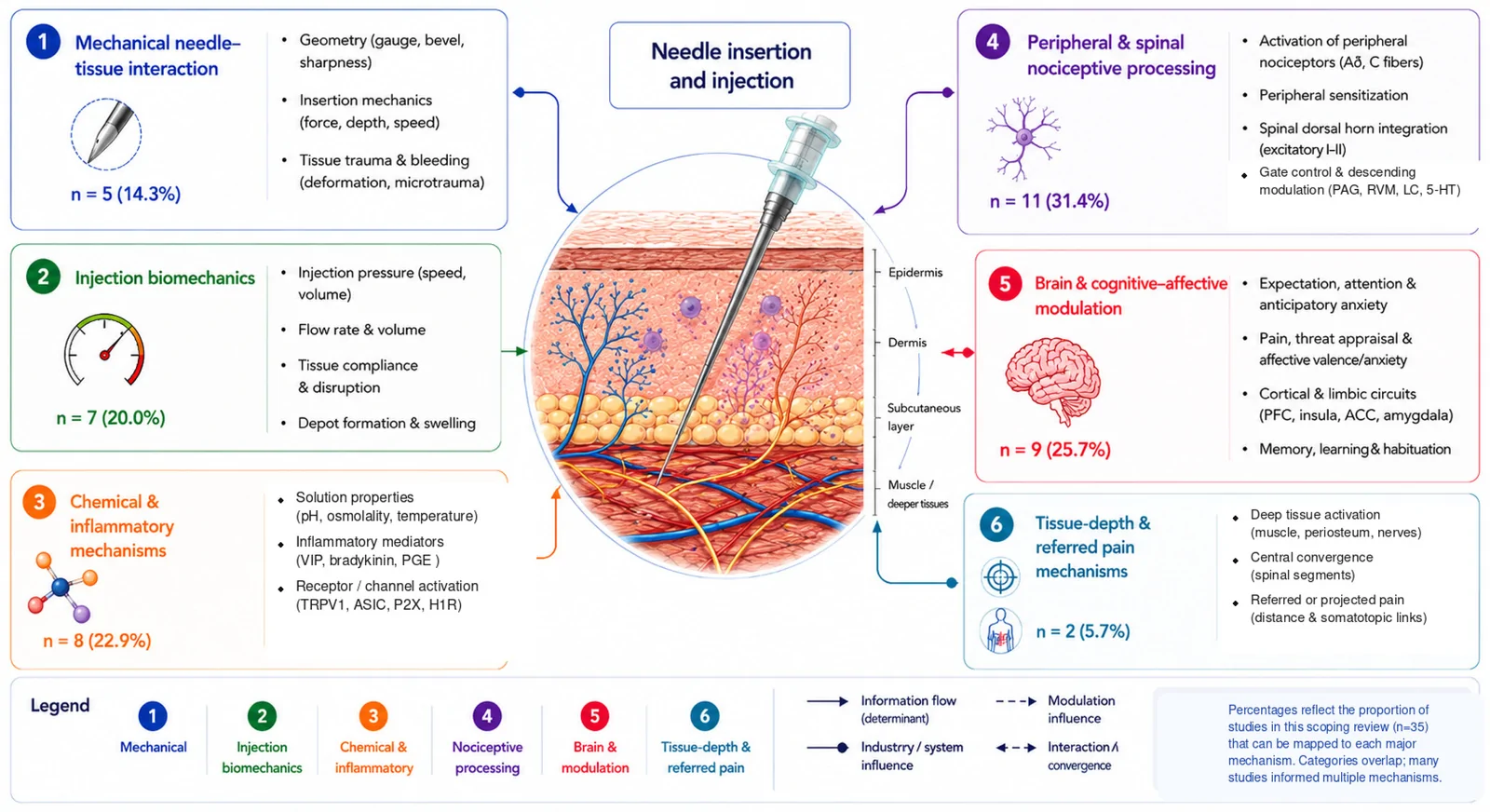
Mechanistic domains contributing to needle-related pain across the included studies.

**Figure 3:**
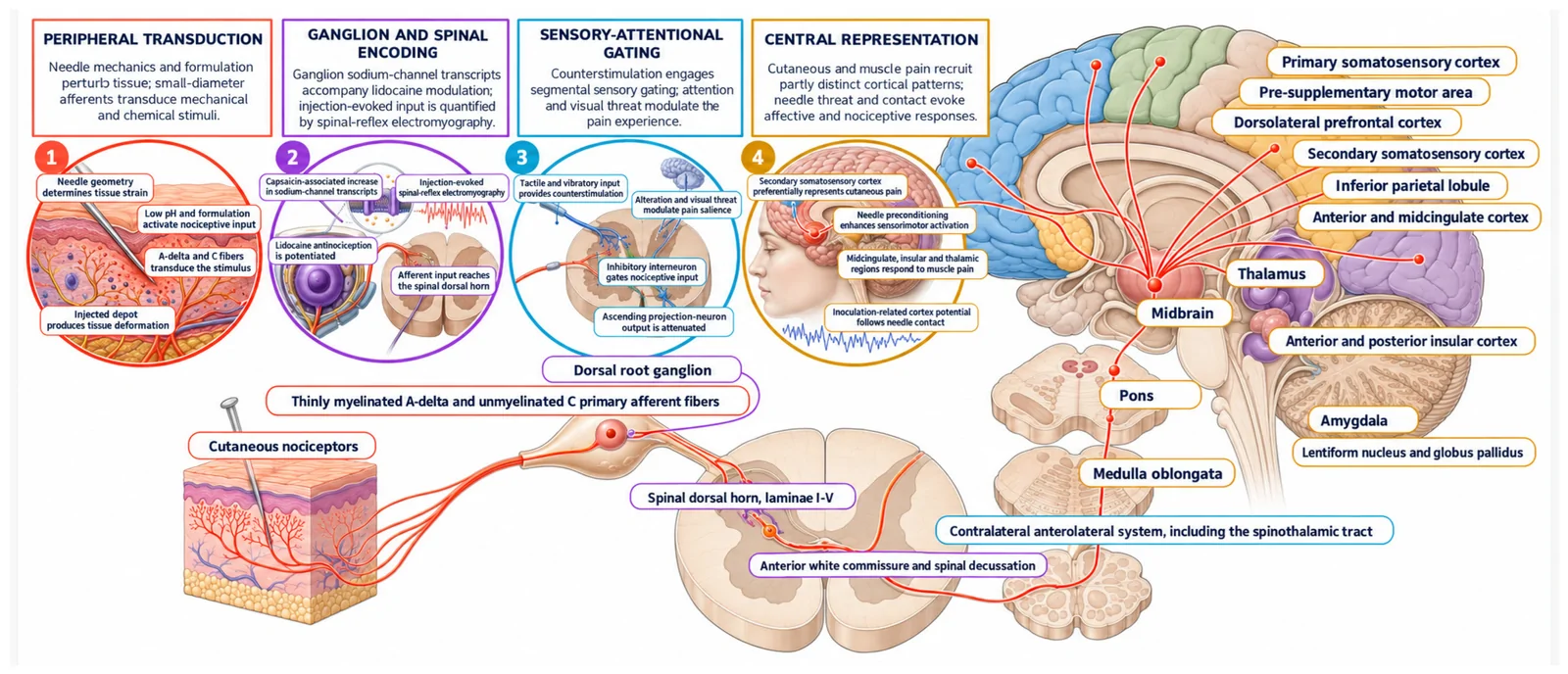
Anatomical and functional integration of mechanistic evidence across the ascending nociceptive pathway. Evidence from the included studies was mapped onto four interconnected mechanistic levels: peripheral transduction [9,11,12,16,26,27,28,29,30,31,32,33,34,35,36,37,38,39], ganglion and spinal encoding [26,28,39], sensory–attentional gating [40,41,42,43,44,45,46,47,48,49,50,51], and central representation [17,18,52,53,54]. The schematic illustrates the progression of nociceptive input from peripheral tissues through ganglionic and spinal processing to supraspinal sensory, attentional, affective, and cortical networks while recognizing modulatory influences at multiple levels.

**Figure 4:**
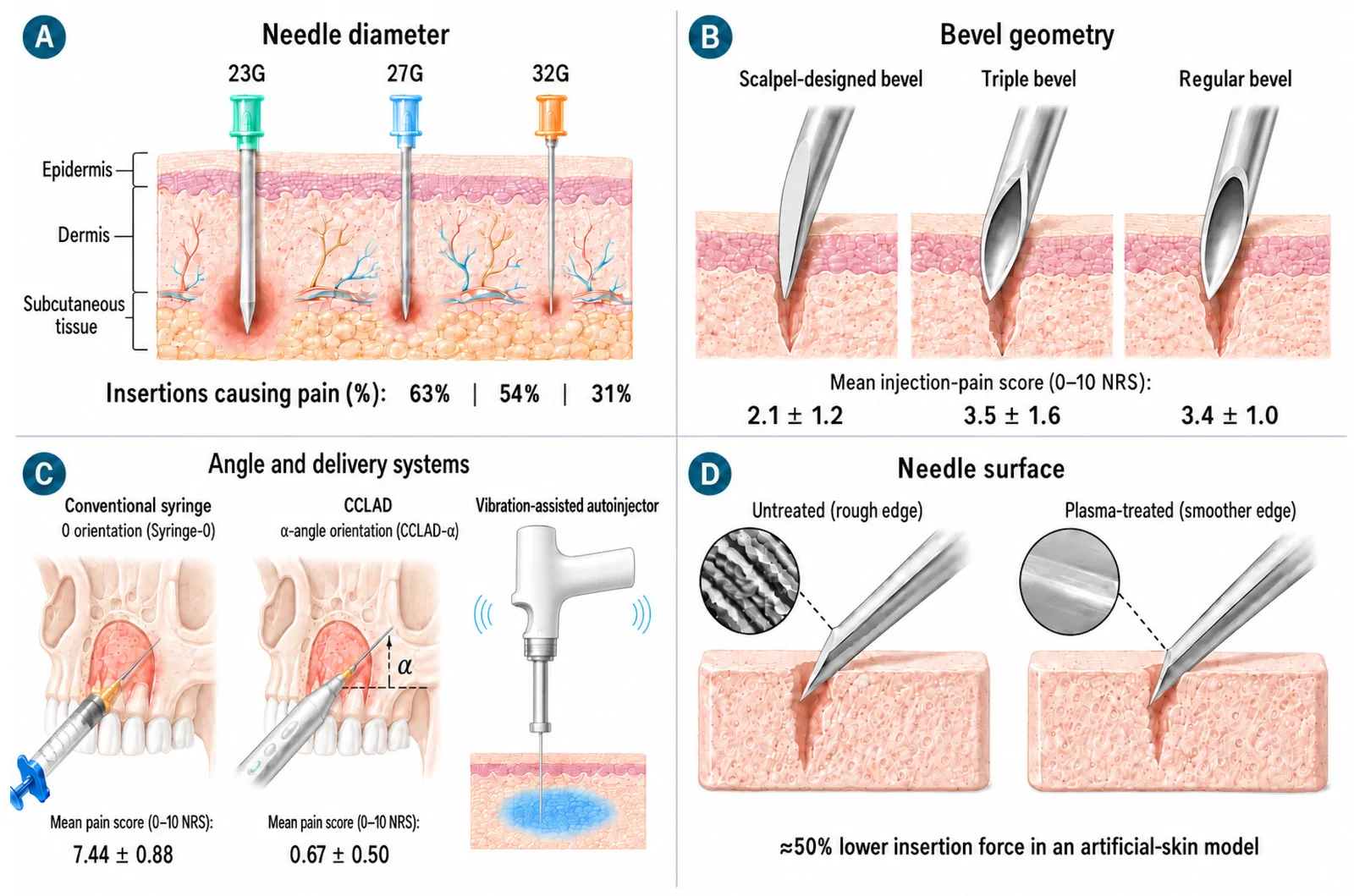
Needle and procedural characteristics represented in studies of mechanical pain during needle insertion. (**A**) Needle diameter and reported frequency of painful insertions [9]. (**B**) Scalpel-designed, triple-bevel, and regular-bevel needles and their reported injection-pain scores [16]. (**C**) Conventional syringe and computer-controlled local anesthetic delivery at different insertion angles [34], together with a schematic of a vibration-assisted autoinjector [55]. (**D**) Untreated and plasma-treated needle surfaces evaluated in an artificial-skin model [27]. G, gauge; CCLAD, computer-controlled local anesthetic delivery; NRS, numerical rating scale. Values are percentages or means ± standard deviations, as indicated.

**Figure 5:**
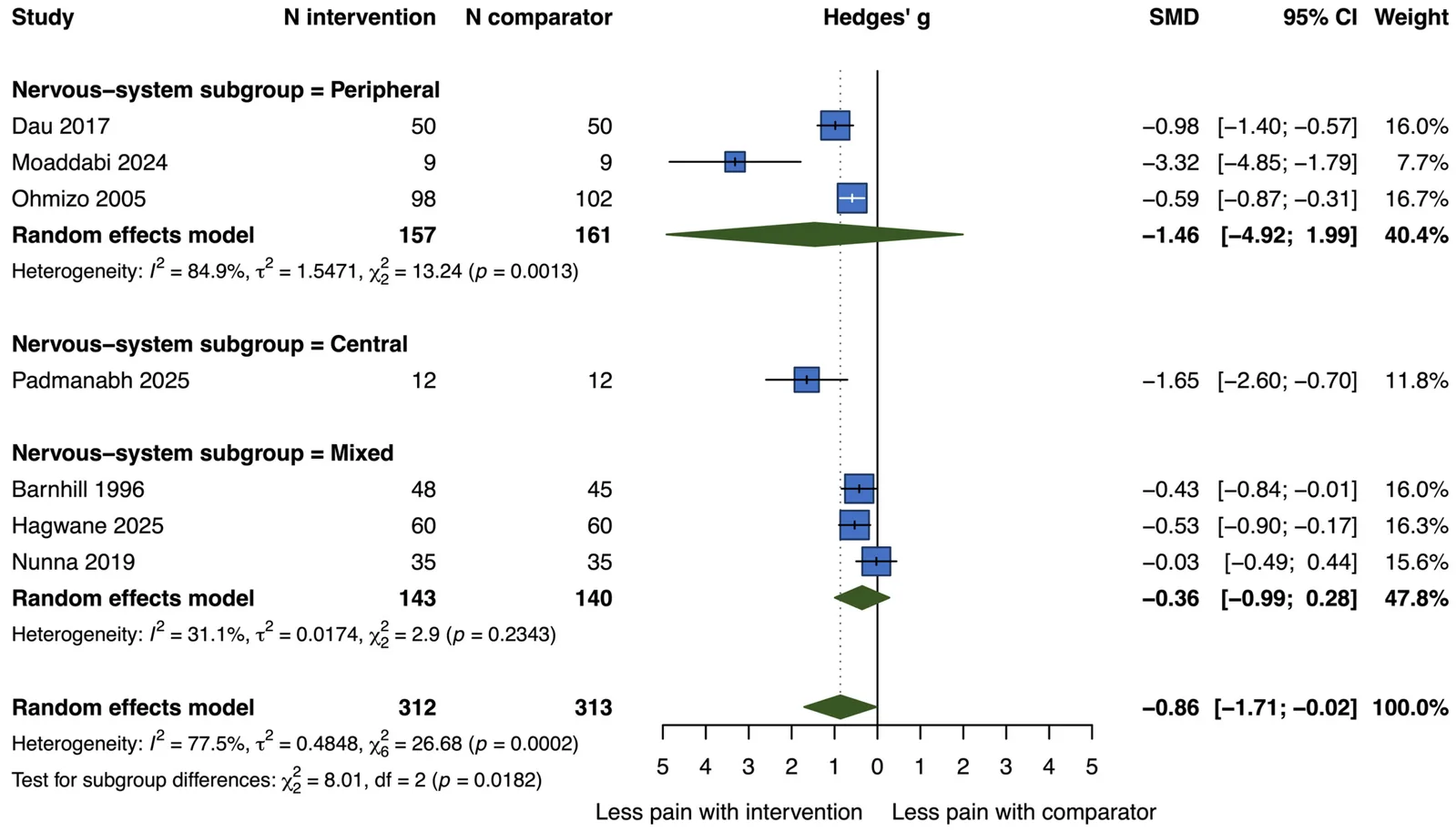
Forest plot of intervention effects on needle-related pain by proposed nervous-system mechanism [16,34,35,42,45,50,54]. Squares represent individual Hedges’ g estimates, with size proportional to statistical weight; horizontal lines indicate 95% confidence intervals. Diamonds represent random-effects pooled estimates for the peripheral and mixed subgroups and for all comparisons combined. The central subgroup contains one comparison and is not pooled. Negative values favor the intervention; the vertical line indicates no difference.

## Data Availability

No new data were created or analyzed in this study. Data sharing is not applicable.

## Supplementary Materials

The following supporting information can be downloaded at https://www.mdpi.com/article/10.3390/medsci14060658/s1, Table S1. Search strategies for PubMed/MEDLINE, Embase, and Web of Science; Table S2. Characteristics of included studies; Table S3. Mechanism classification of included studies; Table S4. Outcomes and mechanistic findings in pre-clinical/non-human studies; Table S5. Outcomes and mechanistic findings in clinical studies; Table S6. Risk-of-bias assessment matrix for the included studies; Table S7. Question key for the risk-of-bias assessment tools. File S1: PRISMA 2020 checklist.

## Abbreviations

ACC	Anterior cingulate cortex
APC	Article processing charge
CCLAD	Computer-controlled local anesthetic delivery
CI	Confidence interval
CNS	Central nervous system
EEG	Electroencephalography
EMG	Electromyography
ETA	Endothelin type A receptor
FLACC	Face, Legs, Activity, Cry, and Consolability
GFAP	Glial fibrillary acidic protein
JBI	Joanna Briggs Institute
LCT	Long-chain triglyceride
MCT	Medium-chain triglyceride
NRS	Numerical rating scale
PAG	Periaqueductal gray
PFC	Prefrontal cortex
PNS	Peripheral nervous system
PRISMA	Preferred Reporting Items for Systematic Reviews and Meta-Analyses
PROSPERO	International Prospective Register of Systematic Reviews
S1	Primary somatosensory cortex
SD	Standard deviation
SEM	Standard error of the mean
SMD	Standardized mean difference
SYRCLE	Systematic Review Centre for Laboratory Animal Experimentation
TRPV1	Transient receptor potential vanilloid 1
VAS	Visual analog scale
VR	Virtual reality
WBFPS	Wong–Baker FACES Pain Rating Scale
