# Supplementary material for: Needle-Related Procedural Pain: An Integrated Nervous-System Framework from Tissue Mechanics to Central Processing—A Systematic Review
Source: Med Sci (Basel). 2026 Oct 10;14(6):658. doi: 10.3390/medsci14060658 (PMC13706745; doi:10.3390/medsci14060658)
Supplement: Supplementary file 1 [file medsci-14-00658-s001.zip › medsci-4563236-supplementary.pdf]

Supplementary Material

Table S1. Search strategies for the online databases PubMed (NCBI), Embase, and Web of Science.

**Pubmed (NCBI)**
(("injection pain"[tiab] OR "needle pain"[tiab] OR "dental injection"[tiab] OR "local anesthesia"[tiab] OR "local anaesthesia"[tiab] OR "pain during injection"[tiab] OR "pain on injection"[tiab]) AND ((mechanism*[tiab] OR nocicept*[tiab] OR nociception[tiab] OR "tissue distension"[tiab] OR "tissue trauma"[tiab] OR "tissue injury"[tiab] OR inflammation[tiab]) OR ("injection pressure"[tiab] OR "flow rate"[tiab] OR "injection volume"[tiab] OR "tissue expansion"[tiab] OR "controlled delivery"[tiab] OR "computer-controlled delivery"[tiab] OR CCLAD[tiab]) OR (vibrat*[tiab] OR vibrotactile[tiab] OR "gate control"[tiab] OR counterstimulation[tiab] OR "sensory modulation"[tiab]))) <br>**Results: 1,475**
**Embase**
(('injection pain':ti,ab OR 'needle pain':ti,ab OR 'dental injection':ti,ab OR 'local anesthesia':ti,ab OR 'local anaesthesia':ti,ab OR 'pain during injection':ti,ab OR 'pain on injection':ti,ab) AND ((mechanism*:ti,ab OR nocicept*:ti,ab OR nociception:ti,ab OR 'tissue distension':ti,ab OR 'tissue trauma':ti,ab OR 'tissue injury':ti,ab OR inflammation:ti,ab) OR ('injection pressure':ti,ab OR 'flow rate':ti,ab OR 'injection volume':ti,ab OR 'tissue expansion':ti,ab OR 'controlled delivery':ti,ab OR 'computer-controlled delivery':ti,ab OR CCLAD:ti,ab) OR (vibrat*:ti,ab OR vibrotactile:ti,ab OR 'gate control':ti,ab OR counterstimulation:ti,ab OR 'sensory modulation':ti,ab))) <br>**Results: 2,766**
**Web of Science**
(("injection pain" OR "needle pain" OR "dental injection" OR "local anesthesia" OR "local anaesthesia" OR "pain during injection" OR "pain on injection") AND ((mechanism* OR nocicept* OR nociception OR "tissue distension" OR "tissue trauma" OR "tissue injury" OR inflammation) OR ("injection pressure" OR "flow rate" OR "injection volume" OR "tissue expansion" OR "controlled delivery" OR "computer-controlled delivery" OR CCLAD) OR (vibrat* OR vibrotactile OR "gate control" OR counterstimulation OR "sensory modulation"))) <br>**Results: 1,646**

Table S2. Characteristics of included studies.

No.	Author (year)	Country	Study design	Study type	Population / model	Sample size	Age	Sex/gender	Needle/procedure context
1	Alfaraj et al. (2022) [26]	Saudi Arabia	In vivo animal study/ RCT	Animal	Female Wistar albino rats	n=32 total; n=16 water/caffeine groups; n=8 per subgroup	Age NR; weight approximately 250 g	Female rats	Intraplantar lidocaine injection after habitual caffeine intake
2	Alghurair et al. (2013) [27]	Japan	Bench experimental study	Model / bench	Artificial skin and medical needles / Polyethylene artificial skin film and 26G stainless-steel needle	Not applicable	Not applicable	Not applicable	Needle insertion into artificial skin
3	Ami et al. (2015) [28]	Japan	Animal experimental study	Animal	Male Crl:CD (SD) rats	n=8 animals per study group	8 weeks old	Male rats	Subcutaneous injection into rat plantar aspect; EMG spinal reflex recording
4	Aminabadi et al. (2008) [40]	Iran	Randomized clinical investigation	Human	Children requiring local anesthesia for primary molar treatment	n=78; three groups n=26 each	4–5 years; mean age 4.72 years	40 male; 38 female	Inferior alveolar nerve block after topical anesthesia
5	Aravena et al. (2018) [29]	Chile	Double-blind split-mouth randomized clinical trial	Human	Adult dental student volunteers	n=72 analyzed	22.1 ± 2.45 years; range 18–29; men 21.9 ± 2.3; women 22.3 ± 2.6	51 men; 21 women	Maxillary buccal infiltration with lidocaine/epinephrine (anterior superior alveolar nerve)
6	Arendt-Nielsen et al. (2006) [9]	Denmark	Controlled experimental study	Human	Healthy volunteers	n=60: Experiment 1: n = 30 healthy volunteers; Experiment 2: n = 30 healthy volunteers	NR	NR	Automated controlled cutaneous needle insertions (23G, 27G, 30G, 32G)
7	Badr et al. (2026) [41]	Syria	Parallel-arm randomized controlled trial	Human	Children undergoing pulpotomy of primary molars	n=112; four equal groups n=28; 14 maxillary and 14 mandibular cases per group	8.13 ± 1.3 years; range 6–10	69 boys (61.6%); 43 girls (38.4%)	Dental local anesthetic administration using different delivery systems
8	Barnhill et al. (1996) [42]	United States	Clinical experimental study	Human	Human participants receiving intramuscular immune globulin injections	n=93: 48 pressure / 45 control	NR (adults ≥18 years)	Pressure: 14 men / 34 womenp; Control: 14 men / 31 women	Intramuscular immune globulin injection with/without manual pressure
9	Berteau et al. (2015) [30]	France	Single-center randomized crossover Phase I study	Human	Healthy adults	n=24	1.1 ± 3.9 years; range 35–50	12 males; 12 females	Abdominal subcutaneous injections using placebo solutions, 27G 6-mm needle
10	Dau et al. (2017) [16]	Germany	Prospective single-blind randomized clinical trial	Human	Dental patients receiving infiltration anesthesia	n=150; three groups n=50 each	Adults >18 years; mean/range NR	NR	Conventional dental infiltration anesthesia
11	Deng et al. (2026) [31]	Australia / China	Multiphysics numerical simulation	Model / simulation	Computational subcutaneous tissue/hydrogel model	Not applicable	Not applicable	Not applicable	Injectable hydrogel depot swelling after injection
12	Edlich et al. (1987) [32]	United States	Experimental performance study	Human + bench	Healthy volunteers and needle/syringe systems	n=8 volunteers for needle-puncture pain; additional injection-	NR	NR	Local anesthesia needle/syringe system evaluation

						depth comparison described in scan			
**13**	Flisfisch et al. (2021) [43]	Switzerland	Prospective randomized split-mouth controlled trial	Human	Adults with tooth-neck defects in different quadrants	n=20	Median 64 years; range 42–76 (mean NR)	10 men; 10 women	Local buccal infiltration anesthesia
**14**	Fowler-Kerry et al. (1987) [53]	Canada	Randomized factorial study	Human	Children receiving routine immunization injections	n=200	4.5–6.5 years	100 boys; 100 girls	Routine immunization injection
**15**	Goodell et al. (2000) [44]	United States	Prospective randomized comparative clinical trial	Human	Endodontic patients undergoing local anesthesia	n=80; 40 per group	19–55 years	Predominantly men; exact counts NR	Dental local anesthetic administration
**16**	Hagwane et al. (2025) [45]	India	Randomized clinical trial; two appointments per child, one without and one with counter-stimulation, separated by 7 days	Human	Healthy cooperative children requiring bilateral maxillary local anesthesia	n=120 total; camouflaged syringe n=60; insulin syringe n=60; no dropouts	8–10 years; insulin 9.07 ± 0.76 years; camouflaged 8.98 ± 0.73 years	Insulin: 55% males / 45% females; camouflaged: 56.7% males / 43.3% females	Bilateral maxillary infiltration anesthesia with camouflaged or insulin syringe; thumb-pressure counter-stimulation adjacent to the injection site when assigned
**17**	Hambouta et al. (2025) [46]	Egypt	Randomized controlled clinical trial, parallel arms	Human	Cooperative children requiring maxillary primary molar extraction	n=48; 12 per subgroup	Buzzy 7.60 ± 1.43; Topical 7.10 ± 1.15 years.	13 boys/11 girls (Buzzy) vs 14 boys/10 girls (Topical)	Maxillary buccal, palatal, and intrapapillary local anesthesia
**18**	Hegde et al. (2019) [47]	India	Randomized crossover split-mouth study	Human	Children requiring bilateral inferior alveolar nerve block	n=30	6–11 years; groups 6–8 and 9–11	15 boys; 15 girls	Inferior alveolar nerve block using conventional vs vibration/distraction device
**19**	Heise et al. (2014) [12]	Germany / Denmark	Single-center one-visit double-blind randomized controlled trial	Human	Adults with type 1 or type 2 diabetes using daily injections	n=82 randomized	54.3 ± 11.9 years; range 22–74	32 female (39%); 50 male (61%)	17 SC saline injections in abdomen/thigh at different speeds/volumes
**20**	Jabeen et al. (2025) [48]	India	Randomized split-mouth controlled trial	Human	Children receiving dental local anesthesia	n=30	5–10 years	NR	Dental local anesthesia with mucosal vibrator vs topical anesthetic
**21**	Jin et al. (2025) [39]	Republic of Korea	In vivo animal experimental study	Animal	Male Sprague-Dawley rats; formalin-induced acute inflammatory pain model	n=15; three groups n=5 each (untreated, general syringe, automatic syringe)	6 weeks old at arrival; 1 week of acclimation before experiments	Male rats	Subcutaneous injection of 50 µl of 2% formalin into the left hind paw with a 30G needle, delivered by a manual syringe or by the I-ject digital automatic syringe
**22**	Karuppiah et al. (2026) [49]	India	Randomized comparative clinical study	Human	Children requiring local infiltration for extraction of primary maxillary teeth	n=45; three groups n=15 each	Total 8.49 ± 1.342 years; range 5–10. Star Pen 8.60 ± 1.298; insulin 8.20 ± 1.568; conventional 8.67 ± 1.175	28 male (62.2%); 17 female (37.8%)	Dental local anesthesia using different syringe/device methods
**23**	Khodorova et al. (2010) [33]	United States	Animal experimental study	Animal	Rat hind paw injection model	n=variable by experiment; commonly n=4–13/group; tachyphylaxis time course n=6–11/group	Adult rat; age NR; 220–250 g	Male rats	Subcutaneous endothelin-1 injection into hind paw footpad

**24**	Kudo et al. (2005) [11]	Japan	Experimental clinical study	Human	Healthy male dental trainees/interns	n=28	26.7 ± 3.6 years	28 men	Oral mucosal injection using computer-controlled local anesthetic delivery system
**25**	Moaddabi et al. (2024) [34]	Iran	Parallel-design randomized clinical trial	Human	Adults needing anterior maxillary labial infiltration	n=36; four groups n=9 each	35.36 ± 12.87 years; range 21–60	22 male (61.1%); 14 female (38.9%)	Labial infiltration anesthesia of anterior maxilla
**26**	Nunna et al. (2019) [50]	India	Prospective randomized single-blind parallel clinical trial	Human	Children requiring LA for pulp therapy or tooth extraction	n=70; CS n=35; VR n=35	CS 8.80 ± 1.389 years; VR 8.91 ± 1.442 years	Overall 35 male / 35 female; CS 45.7% boys/54.3% girls; VR 54.3% boys/45.7% girls	Dental local anesthesia administration
**27**	Ohmizo et al. (2005) [35]	Japan	Double-blind randomized clinical study plus laboratory assay	Human	Adult surgical/anesthesia patients; healthy volunteer blood samples	n=200 patients: LCT n=102, LCT/MCT n=98; n=13 healthy volunteers for blood assay	Clinical age: LCT 53±16 (18–83); LCT/MCT 49±16 (18–83). Volunteers 32±7 (21–48)	Clinical: LCT 35M/67F; LCT/MCT 45M/53F; volunteers 7M/6F	Intravenous propofol injection
**28**	Padmanabh et al. (2025) [54]	India	Randomized controlled study	Human	Children receiving dental local anesthesia	n=36; three groups n=12	7–11 years	NR	Dental local anesthesia administration
**29**	Rukwied et al. (2007) [36]	Germany	Psychophysical experimental study	Human	Healthy male subjects	n=16	32 ± 12 years	16 male	Microinjections of protons/PGE2 into forearm skin or anterior tibial muscle
**30**	Sago et al. (2023) [51]	Denmark	Repeated-session experimental study	Human	Healthy participants	n=50 assessed; 52 recruited	>18 years; exact mean NR	26 men; 26 women recruited	Masseter palpation; conditioned pain modulation; local anesthetic/saline masseter injections
**31**	Udagawa et al. (2018) [37]	Denmark	Randomized experimental injection study	Human	Healthy volunteers	n=16	24.7 ± 4.0 years	12 men; 4 women	Glutamate/saline injection into masseter-region tissues at different depths
**32**	Uematsu et al. (2011) [52]	Japan	fMRI experimental study	Human	Healthy volunteers	n=17	23–33 years	10 male; 7 female	Tonic pressure to calf with/without local anesthesia to isolate muscle vs cutaneous pain
**33**	Ushida et al. (2008) [18]	Japan	fMRI experimental study with needle preconditioning subgroup	Human	Healthy right-handed volunteers	n=30; preconditioned n=15; non-preconditioned n=15	Overall mean 29 years, range 22–34; preconditioned mean 26.9 years	Overall 16 male/14 female; preconditioned 9 male/6 female	Viewing virtual video of needle puncturing palm; real needle puncture preconditioning in one subgroup
**34**	Verriotis et al. (2015) [17]	United Kingdom	Observational EEG study during routine inoculation	Human	Healthy term-born infants	n=15 infants; 1–2 months n=12; 12 months n=5; some repeated/excluded inoculations described	1–2 months and 12 months	1–2 months: 6/12 female; 12 months: 3/5 female	Routine inoculation needle prick
**35**	Zijlstra et al. (2018) [38]	Germany	Clinical experimental study	Human	Adults with type 1 or type 2 diabetes receiving daily injections	n=80 included; 79 in full pain analysis; 24 injections each	Adult; exact age NR	Male and female adults; exact counts NR	SC saline injections into abdomen/thigh with varying volume, speed, site

Crl:CD(SD), Charles River CD Sprague-Dawley rat strain; EMG, electromyography; G, gauge; LA, local anesthesia; LCT, long-chain triglyceride; LCT/MCT, long-chain triglyceride/medium-chain triglyceride; M/F, male/female; n, sample size; NA, not applicable; NR, not reported; PGE2, prostaglandin E2; SC, subcutaneous; SD, standard deviation; VR, virtual reality.

Table S3. Mechanism classification of included studies.

No.	Author (year)	Nervous system level	Primary mechanism category	Mechanism subtype	Exposure/intervention	Comparator/control
**1**	Alfaraj et al. (2022) [26]	Peripheral	Chemical / formulation / receptor mechanisms/ Voltage-gated sodium channels	Lidocaine action; chronic caffeine; Nav1.3/Nav1.7/Nav1.8 sodium channels	Habitual caffeine intake + lidocaine	Control, lidocaine alone, caffeine alone
**2**	Alghurair et al. (2013) [27]	Peripheral	Mechanical puncture / needle design	Needle surface roughness; cutting-edge sharpness; insertion force	Ion-plasma sputtering / plasma-etched needles	Untreated needles
**3**	Ami et al. (2015) [28]	Peripheral	Chemical / formulation factors	pH; injection volume; osmotic pressure; biologic formulation; spinal reflex EMG	Solutions varying by injection volume, pH, osmotic pressure, and biologic drug formulation	Different formulation conditions
**4**	Aminabadi et al. (2008) [40]	Mixed CNS–PNS	Psychological and sensory-modulatory mechanisms	Pressure/vibration; verbal distraction; gate-control and attention mechanisms	Pressure/vibration alone or Pressure/vibration + verbal distraction	Simple anesthesia control
**5**	Aravena et al. (2018) [29]	Peripheral	Chemical / formulation factors	Warmed anesthetic; temperature-dependent lidocaine action; TRPV1/nociceptor permeability	Lidocaine warmed to 42°C	Room-temperature lidocaine (21°C)
**6**	Arendt-Nielsen et al. (2006) [9]	Peripheral	Mechanical puncture / needle design	Outer needle diameter; bleeding; skin trauma	Needles with different outer diameters	Different needle gauges
**7**	Badr et al. (2026) [41]	Mixed CNS–PNS	Psychological and sensory-modulatory mechanisms	Device-controlled injection mechanics; vibration (gate-control); needle-free delivery; anxiety/autonomic modulation	CCLAD, vibration-assisted injection, needle-free jet injection	Conventional manual syringe
**8**	Barnhill et al. (1996) [42]	Mixed CNS–PNS	sensory-modulatory mechanisms	Manual pressure; tactile counterstimulation; gate-control mechanism	Manual pressure applied to injection site for 10 seconds before injection	No pressure / standard injection
**9**	Berteau et al. (2015) [30]	Peripheral	Injection mechanics / formulation properties	Viscosity; injection volume; flow rate; subcutaneous depot location	2 or 3 mL injections; viscosities 1, 8–10, 15–20 cP; flow rates 0.02 or 0.3 mL/s	Within-participant injection conditions
**10**	Dau et al. (2017) [16]	Peripheral	Mechanical puncture / needle design	Needle bevel design; needle deformation; barbs/soft-tissue trauma	Scalpel-designed bevel needle	Triple-bevel and regular-bevel needles
**11**	Deng et al. (2026) [31]	Peripheral	Swelling-induced tissue mechanics / tissue distension	Post-injection swelling; poro-viscoelastic tissue stress; depot geometry	Hydrogel swelling parameters; injection depth; depot radius/volume	Different simulated injection depths and hydrogel radii/volumes.
**12**	Edlich et al. (1987) [32]	Peripheral	Mechanical puncture / needle design	Needle gauge; bending force; fluid dynamics; puncture pain	25G, 27G, 30G needles and syringe configurations	Different needle gauges/syringes
**13**	Flisfisch et al. (2021) [43]	Mixed CNS–PNS	Injection mechanics and psychological mechanisms	Computer-controlled local anesthetic delivery (CCLAD); flow-rate control; device anxiety/comfort	CCLAD	Conventional syringe
**14**	Fowler-Kerry et al. (1987) [53]	Central	Central cortical / affective / attention mechanisms	Music distraction; suggestion; attentional pain modulation	Music distraction and/or suggestion	Control
**15**	Goodell et al. (2000) [44]	Mixed CNS–PNS	Injection mechanics and psychological mechanisms	Controlled-rate injection system / Wand; pressure/flow; anxiety and tolerance	Controlled injection pressure system (Wand)	Conventional atraumatic syringe technique
**16**	Hagwane et al. (2025) [45]	Mixed CNS–PNS	Psychological and sensory-modulatory mechanisms	Syringe visual threat/camouflage; anxiety reduction; thumb-pressure counter-stimulation; gate-control mechanism	Camouflaged syringe and/or thumb-pressure counter-stimulation.	Insulin syringe (Dispovan) with and without thumb-pressure counter-stimulation
**17**	Hambouta et al. (2025) [46]	Mixed CNS–PNS	Psychological and sensory-modulatory mechanisms	Cold vibration (Buzzy); topical gel; palatal vs intrapapillary injection; gate control	Buzzy® external cold/vibration for 2 min	Topical benzocaine gel; palatal vs intrapapillary techniques
**18**	Hegde et al. (2019) [47]	Mixed CNS–PNS	Psychological and sensory-modulatory mechanisms	Vibration plus distraction; gate control; anxiety reduction	Child-friendly vibration/distraction device	Conventional injection method

**19**	Heise et al. (2014) [12]	Peripheral	Injection pressure / volume / tissue distension	Injection volume; speed; injection site	Different SC injection speeds (150, 300, 450 μL/s) and volumes (400–1600 μL)	Needle-only insertions and within-person conditions
**20**	Jabeen et al. (2025) [48]	Mixed CNS–PNS	Psychological and sensory-modulatory mechanisms	Mucosal vibration; gate control; topical benzocaine comparator	Custom-designed mucosal vibrator	Topical benzocaine gel
**21**	Jin et al. (2025) [39]	Peripheral	Injection mechanics / device-controlled delivery	Automated control of injection pressure, speed and volume; reduced mechanical tissue trauma; spinal neuronal activation (c-fos) and astroglial activation (glial fibrillary acidic protein)	I-ject™ digital automatic syringe (programmable flow rate, volume and speed)	Conventional manual disposable syringe with an identical 30G needle; untreated (no injection) group
**22**	Karuppiah et al. (2026) [49]	Mixed CNS–PNS	Psychological and sensory-modulatory mechanisms	Star Pen; insulin syringe; conventional syringe; device appearance and delivery control	Star Pen device; insulin syringe	Conventional syringe
**23**	Khodorova et al. (2010) [33]	Peripheral	Chemical / receptor mechanisms	Endothelin-1; ETA receptor sensitivity; peripheral receptor tachyphylaxis	Repeated ET-1 injections; sciatic nerve local anesthesia; naloxone; BQ-123	Repeated injections and pharmacologic controls
**24**	Kudo et al. (2005) [11]	Peripheral	Injection pressure / volume / tissue distension	Initial injection pressure; tissue distension; anxiety response	Low-speed vs high-speed injection; real-time pressure measurement	Different injection speeds/pressures
**25**	Moaddabi et al. (2024) [34]	Peripheral	Mechanical puncture	Needle insertion angle; CCLAD; tissue entry geometry	Needle at alpha angle ± CCLAD	Parallel/tooth-axis orientation; conventional syringe
**26**	Nunna et al. (2019) [50]	Mixed CNS–PNS	Psychological and sensory-modulatory mechanisms	VR distraction versus counter-stimulation; attention/anxiety/gate control	VR distraction with ANTVR glasses	Counter-stimulation
**27**	Ohmizo et al. (2005) [35]	Peripheral	Chemical / formulation / receptor mechanisms	Propofol formulation; aqueous-phase propofol concentration; bradykinin; complement activation	LCT/MCT propofol	LCT propofol
**28**	Padmanabh et al. (2025) [54]	Central	Central cortical / affective / attention mechanisms	Audiovisual distraction; smart glasses; VR; anxiety and attention	Smart glasses or VR glasses	Tell-show-do/control condition
**29**	Rukwied et al. (2007) [36]	Peripheral	Chemical / formulation / receptor mechanisms	Low pH/protons; PGE2; TRPV1/ASIC sensitization; inflammatory pain	Low pH solutions ± PGE2	PGE2 alone; saline; skin vs muscle
**30**	Sago et al. (2023) [51]	Mixed CNS–PNS	Tissue-depth / referred-pain mechanisms	Referred sensation; WDR/convergence; descending inhibition; peripheral afferent block	CPM painful conditioning; local anesthetic masseter block	Baseline/saline
**31**	Udagawa et al. (2018) [37]	Peripheral	Chemical / formulation / receptor mechanisms	Glutamate-evoked pain; tissue depth; NMDA-related mechanisms; pressure sensitization	Glutamate injection into subcutaneous, intramuscular, or bone-surface tissues	Isotonic saline; tissue depths
**32**	Uematsu et al. (2011) [52]	Mixed CNS–PNS	Tissue-depth / central pain processing mechanisms	Cutaneous versus muscle pain; deep-tissue pain network; central processing pattern	Painful/non-painful pressure with/without local anesthesia	Skin + muscle vs isolated muscle stimulation conditions
**33**	Ushida et al. (2008) [18]	Central	Central cortical / affective / attention mechanisms	Visual threat; anticipation; unpleasant memory retrieval; pain/emotion networks	Virtual needle puncture; real needle preconditioning	Non-preconditioned group
**34**	Verriotis et al. (2015) [17]	Central	Central cortical / affective / attention mechanisms	Infant cortical nociceptive processing; developmental EEG response	Needle contact during inoculation	Age groups
**35**	Zijlstra et al. (2018) [38]	Peripheral	Injection pressure / volume / tissue distension	Injection volume; speed; anatomical site	Different volumes (0–2250 μL), speeds (0–800 μL/s), and injection sites	Within-subject injection conditions; needle-only insertions

ANTVR, ant virtual reality device/glasses; ASIC, acid-sensing ion channel; BQ-123, endothelin-A receptor antagonist; CCLAD, computer-controlled local anesthetic delivery; CNS, central nervous system; CPM, conditioned pain modulation; cP, centipoise; CS, counter-stimulation; EMG, electromyography; ET-1, endothelin-1; ETA, endothelin-A receptor; LA, local anesthesia; LCT, long-chain triglyceride; LCT/MCT, long-chain triglyceride/medium-chain triglyceride; Nav, voltage-gated sodium channel; NMDA, N-methyl-D-aspartate; PGE2, prostaglandin E2; PNS, peripheral nervous system; SC, subcutaneous; TRPV1, transient receptor potential vanilloid 1; VR, virtual reality; WDR, wide dynamic range.

Table S4. Outcomes and mechanistic findings in pre-clinical/non-human studies.

No.	Author (year)	Population / model	Pain outcome measures	Behavioral or physiological outcomes	Key results	Mechanistic conclusion
**1**	Alfaraj et al. (2022) [26]	Female Wistar albino rats	Paw pressure test; paw withdrawal latency	mRNA expression of Nav1.3, Nav1.7, Nav1.8 in dorsal root ganglia	LIDO+CAF reduced pressure sensation vs control and showed stronger effect than LIDO alone; synergistic effects at 30 and 60 min; thermal pain-relieving effects increased; caffeine upregulated Nav1.3/Nav1.7/Nav1.8.	Chronic caffeine consumption potentiated the local anesthetic effect of lidocaine, suggesting peripheral sodium-channel modulation of local anesthetic efficacy.
**2**	Alghurair et al. (2013) [27]	Artificial skin	Insertion force (surrogate for tissue trauma/pain)	Microscopic observation of artificial skin deformation and force-distribution dynamics	Plasma-treated needles reduced insertion force by approximately half versus untreated needles; sharp force-drop events at cutting-face corners were decreased.	Lower cutting-edge roughness and sharper edges reduce insertion force, supporting needle surface mechanics as a peripheral mechanical pain mechanism.
**3**	Ami et al. (2015) [28	Male rats	EMG magnitude as nociceptive spinal reflex	Spinal reflex EMG amplitude (physiologic neurophysiologic measure)	EMG responses increased with injection volume and acidity; responses did not depend on osmotic pressure up to osmotic ratio approximately 5; adalimumab induced approximately twice the EMG magnitude of etanercept.	Injection pain can be quantified by spinal reflex EMG and is driven by volume, and acidity/formulation more than osmotic pressure in this model, more strongly influenced by injection volume and acidity/formulation than by osmolarity below an osmotic ratio of ~5
**11**	Deng et al. (2026) [31]	Computational subcutaneous tissue/hydrogel model	Peak tissue stress relative to nociceptor threshold	Von Mises stress, tissue displacement, radial strain, volumetric strain	Simulated tissue stress peaked at approximately 60–100 min after injection, reaching ~10.8 kPa, above reported nociceptor activation threshold (~6–9 kPa); equilibrium after ~400 min. Optimal depth window 6–12 mm; smaller radius/volume could increase local peak stress.	Delayed pain after injectable hydrogels may arise from swelling-induced tissue stress and mechanonociceptor activation.
**21**	Jin et al. (2025) [39]	Male Sprague-Dawley rats	Duration of limping and frequency of licking/biting of the injected paw, over the first 15 min and over the full 60 min	Spinal cord (L4-L5) mRNA levels of c-fos, glial fibrillary acidic protein (GFAP), Oprl1, Htr2a, Tacr1 and Oprm1 by quantitative real-time PCR	First 15 min: limping 553.2 s with the manual syringe versus 383.8 s with the automatic syringe, a 30.6% reduction ($P>0.05$); licking 10.0 versus 3.4 episodes, a 66.0% reduction ($P<0.05$). Over 60 min: limping −2.6% and licking −19.5% (both $P>0.05$). Automatic versus manual syringe: c-fos −29.7% ($P<0.05$), GFAP −81.8% ($P<0.001$), Oprl1 −49.6% ($P<0.001$), Htr2a −16.6% ($P<0.01$) and Oprm1 −56.27% ($P<0.001$); Tacr1 did not differ between groups ($P>0.05$).	Mechanically controlled, device-delivered injection reduced early nocifensive behaviour and attenuated spinal neuronal and astroglial activation, indicating that injection mechanics modulate both peripheral nociceptive input and spinal amplification of pain.
**23**	Khodorova et al. (2010) [33]	Male rats	Nocifensive flinching/ von Frey paw withdrawal threshold.	Not applicable	Second ET-1 flinching response was 20±5%, 57±18%, 79±35%, and 100±17% of first response at 24, 30, 48, and 72 h. Sciatic local anesthesia and naloxone did not prevent tachyphylaxis; local ETA blockade reduced tachyphylaxis.	ET-1 injection pain tachyphylaxis is peripheral, depending on ETA receptor sensitivity/availability rather than reduced CNS responsiveness.

CAF, caffeine; DRG, dorsal root ganglia; EMG, electromyography; ET-1, endothelin-1; ETA, endothelin-A receptor; kPa, kilopascal; LIDO, lidocaine; mRNA, messenger RNA; NA, not applicable; Nav, voltage-gated sodium channel; P, probability value; PNS, peripheral nervous system; SD, standard deviation.

Table S5. Outcomes and mechanistic findings in clinical studies.

No.	Author (year)	Pain outcome measures	Behavioral or physiological outcomes	Key results	Mechanistic conclusion

**4**	Aminabadi et al. (2008) [40]	Sound–Eye–Motor (SEM) pain reaction scale	Behavioral pain reaction	SEM values: simple anesthesia 8.25; counterstimulation 5.07; counterstimulation + distraction 3.41. Simple anesthesia had significantly higher pain reaction than the other groups (P<0.05); combined technique was significantly better than counterstimulation alone.	Counterstimulation likely reduces the local sensory component of pain, while distraction reduces the central/attentional component.
**5**	Aravena et al. (2018) [29]	100-mm VAS pain during injection	NA	Room-temperature anesthesia VAS 35.3 ± 16.71 mm; 42°C anesthesia VAS 15.0 ± 14.67 mm; P<0.001.	Warming local anesthetic significantly reduced dental injection pain, possibly by improving anesthetic entry through TRPV1-related mechanisms.
**6**	Arendt-Nielsen et al. (2006) [9]	Pain frequency; VAS pain intensity; bleeding occurrence	NA	Painful insertion frequency increased with larger diameter: 23G 63%, 27G 54%, 32G 31%; P<0.0001. Bleeding insertions were approximately 1.3 times more painful than non-bleeding insertions (P=0.004).	Thinner needles reduce frequency of needle insertion pain and bleeding, supporting a peripheral puncture/skin trauma mechanism.
**7**	Badr et al. (2026) [41]	Wong–Baker Faces Pain Rating Scale; FLACC	Venham Picture Test; heart rate; oxygen saturation; Houpt cooperation; parental satisfaction	Manual syringe had higher Wong-Baker pain, FLACC distress, and Venham anxiety during anesthesia vs alternative techniques (P≤0.05 for key comparisons). CCLAD showed the most consistent benefit; SpO2 stable; parental satisfaction higher with alternatives (P≤0.05).	Technique-dependent injection distress is influenced by both tissue-delivery mechanics and central anxiety/behavioral response; CCLAD most consistently reduced pain/anxiety.
**8**	Barnhill et al. (1996) [42]	Self-reported injection pain	NA	Study tested whether pre-injection manual pressure reduced IM injection pain; uploaded scan indicates pressure intervention versus control, but exact pain values were not reliably extracted.	Pressure may reduce pain through activation of large-diameter afferents and spinal gating before nociceptive input from IM injection.
**9**	Berteau et al. (2015) [30]	100-mm VAS pain	None; 2D ultrasound assessment of injection depot	Viscosity affected pain (P=0.0003): high viscosity VAS 12.6 mm, medium 16.6 mm, low 22.1 mm; high vs low P=0.0002. Volume (2 vs 3 mL) P=0.89 and flow rate P=0.79 were not significant. 92% of injections were exclusively SC.	Within tested ranges, volume and flow did not drive pain; formulation/tissue distribution factors may be more important than expected.
**10**	Dau et al. (2017) [16]	Numeric rating scale pain; SEM assessment of needle-tip deformation	NA	Injection pain was lower with scalpel bevel: SB 2.1 ± 1.2 vs TB 3.5 ± 1.6 and RB 3.4 ± 1.0; all P<0.001. Deformation detected in 97.3% of needles; barbs: SB 29/50, TB 17/50, RB 19/50.	Needle bevel geometry influences puncture pain; post-injection needle-tip deformation/barbs may contribute to tissue trauma.
**12**	Edlich et al. (1987) [32]	Needle-puncture pain (0-3 ordinal scale)	Needle bending force and fluid-dynamic measurements	30G needles caused less puncture pain than larger needles; flow rate decreased with larger syringes and smaller internal needle diameter; authors recommended 30G/10 mL for infiltration and slow injection over ~10 s.	Needle gauge and syringe/flow dynamics influence peripheral mechanical pain and injection comfort.
**13**	Flisfisch et al. (2021) [43]	VAS: mucosal puncture sensation, pain during administration, pain during treatment.	VAS visual anxiety-inducement; duration of numbness	Anxiety-inducement and pain during administration were approximately three times higher with conventional syringe than CCLAD: 35.95% vs 11.85%, P<0.001; 21.3% vs 7.7%, P=0.005. No difference in mucosal puncture sensation or treatment pain; preference shifted toward CCLAD over time (P=0.01).	CCLAD improved comfort mainly by reducing administration pain and visual/anxiety burden, linking delivery control to pain experience.
**14**	Fowler-Kerry et al. (1987) [53]	4-point child pain scale	NA	Distraction significantly decreased pain; suggestion did not; combining suggestion with distraction did not improve beyond distraction alone. Age influenced distraction success and pain reporting. Control 1.78; Suggestion 1.59; Distraction 1.34; Distraction + Suggestion 1.07 (p=0.007)	Attention/distraction can reduce injection pain in children, supporting a central psychological modulation mechanism.
**15**	Goodell et al. (2000) [44]	VAS pain during injection	Pre/post dental injection anxiety; anticipated future injection anxiety; overall injection experience (VAS).	Patients had significantly lower post-injection anxiety, lower injection pain, and more positive overall experience with conventional atraumatic syringe than controlled-rate system. Table values showed reductions in anxiety questions in both groups, but conventional was superior overall.	A controlled delivery device does not guarantee less pain; operator technique, expectations, and device-related anxiety may influence outcomes.
**16**	Hagwane et al. (2025) [45]	Wong-Baker Faces Pain Rating Scale	Pulse rate; FLACC behavioral scale	Groups were comparable for age (P=0.539) and sex distribution (P=0.854). First appointment pain scores were lower with camouflaged syringe than insulin syringe before treatment (5.83 ± 0.76 vs 6.45 ± 1.24; P=0.001) and after treatment (3.58 ± 0.74 vs 4.17 ± 1.37; P=0.005). Heart rate differed significantly at first appointment before and after treatment (P<0.001). FLACC values in Table 3 show first-appointment post-treatment insulin 2.27 ± 0.78 vs camouflaged 2.80 ±	Camouflaged syringes reduce the visual threat of the injection and anxiety-related amplification of pain; thumb-pressure counter-stimulation provides tactile input consistent with spinal gate-control modulation.

				0.68 (P<0.001), while the abstract/text conclude better behavioral responses with camouflaged syringes. Counter-stimulation further enhanced effects.	
**17**	Hambouta et al. (2025) [46]	VAS pain; SEM behavior	NA	No statistically significant difference between Buzzy and topical gel for buccal anesthesia; significant subjective/objective pain reductions with Buzzy for palatal and intrapapillary anesthesia comparisons.	Cold/vibratory stimulation can reduce pain through peripheral sensory modulation and spinal gating, especially for palatal/intrapapillary anesthesia.
**18**	Hegde et al. (2019) [47]	Wong-Baker Pain Rating Scale; FLACC	Pulse rate before/during injection	Device method produced lower mean pulse rate, FLACC scores, and Wong-Baker pain scores than conventional method.	Vibration/distraction reduces needle pain through sensory gating and attentional/anxiety mechanisms.
**19**	Heise et al. (2014) [12]	100-mm VAS pain; pain acceptability	NA	Injection speed had no effect (P=0.833). Larger volumes increased pain: 1600 vs 400 μL LS mean difference 7.2 mm (95% CI 4.6–9.7; P<0.0001); 1600 vs 800 μL 7.2 mm (4.4–10.0; P<0.0001). Thigh more painful than abdomen by 9.0 mm (6.7–11.3; P<0.0001).	SC injection pain is more strongly related to volume and anatomical site than speed within the tested range.
**20**	Jabeen et al. (2025) [48]	Wong-Baker FACES; Sound–Eye–Motor scale	Pulse rate	Mucosal vibrator significantly reduced subjective and objective pain scores compared with benzocaine gel (P<0.05). Pulse rate changes were not significantly different: vibrator baseline 89.20 ± 8.54, post 89.87 ± 9.11 (P=0.349); benzocaine baseline 89.20 ± 8.54, post 90.60 ± 8.92 (P=0.057).	Mucosal vibration reduced pain without clear anxiety/physiologic change, consistent with local sensory gating/distraction.
**22**	Karuppiah et al. (2026) [49]	Animated Emoji Scale; FLACC	Pulse rate; SpO2	Pain/anxiety were significantly lower in Star Pen and insulin syringe groups than conventional. Animated Emoji before/after: Star Pen 1.27±0.458 to 2.67±0.816; insulin 1.13±0.352 to 2.73±0.799; conventional 1.33±0.617 to 3.00±0.655. Pulse rates differed before (P=0.004) and during (P=0.002).	Smaller/less threatening devices and controlled delivery may reduce pediatric injection pain and anxiety through sensory and psychological mechanisms.
**24**	Kudo et al. (2005) [11]	VAS pain	Faces Anxiety Scale; trait anxiety	Injection pressure correlated with pain (rs=0.579, P=0.00124) and state anxiety (rs=0.479, P=0.00979). Pressure ranged 33–496 mmHg; VAS pain 0–86. Authors recommended pressure <306 mmHg.	Higher injection pressure increases pain and anxiety, supporting tissue distension/pressure as a peripheral driver with affective consequences.
**25**	Moaddabi et al. (2024) [34]	NRS pain	Heart rate; blood pressure; respiratory rate	Mean pain scores: Syringe-0 7.44±0.88; Syringe-alpha 4.67±0.70; CCLAD-0 2.89±0.60; CCLAD-alpha 0.67±0.50; P<0.001. Age and sex not significant (P=0.914 and P=0.702); vital signs not significantly different.	Needle insertion angle and CCLAD reduced pain, supporting a mechanical/procedural modulation of nociceptive input.
**26**	Nunna et al. (2019) [50]	Wong-Baker Faces; VAS	VCARS; pulse rate before/during/after LA	WBFPS: CS 2.97±2.491 vs VR 3.03±2.022, P=0.770. VAS: CS 3.06±2.313 vs VR 2.94±1.765, P=0.991. Pulse during: CS 101.67±17.346 vs VR 93.34±14.353, P=0.032; after: CS 100.51±15.399 vs VR 91.56±12.679, P=0.010. VCARS improved significantly within VR group (P=0.008).	VR reduced injection anxiety more than counter-stimulation, while pain ratings were similar between groups.
**27**	Ohmizo et al. (2005) [35]	Injection pain score 0–4	Bradykinin and activated complement C3a concentrations	Pain scores: LCT 0/1/2/3/4 = 39(38.2%) / 16(15.7%) / 21(20.6%) / 18(17.7%) / 8(7.8%); LCT/MCT = 62(63.3%) / 13(13.3%) / 15(15.3%) / 7(7.1%) / 1(1.0%); P<0.01. Bradykinin/C3a elevated with both propofols vs saline without explaining group difference.	Reduced injection pain with LCT/MCT likely relates to lower aqueous-phase propofol concentration rather than bradykinin or complement differences.
**28**	Padmanabh et al. (2025) [54]	VAS pain/anxiety scores	Pulse rate	Before/after values: Group I VAS 6.58±0.996 to 4.67±0.779 (P=0.001), pulse 98.92±1.93 to 90.42±5.84 (P=0.020). Group II VAS 6.50±1.567 to 4.42±0.669 (P<0.001), pulse 100.17±2.33 to 92.50±4.60 (P=0.028). Group III VAS 6.42±1.379 to 6.00±1.128 (P=0.209), pulse 101.58±3.53 to 99.17±4.41 (P=0.05).	Audiovisual/VR distraction reduced pain/anxiety more than control, consistent with attentional and affective modulation.
**29**	Rukwied et al. (2007) [36]	Pain intensity; heat pain thresholds; pressure pain thresholds; axon-reflex erythema	NA	Low pH caused moderate pain within 5 s and declined over 15–20 s. PGE2 + low pH produced significantly increased second-phase pain at 20–100 s in skin and muscle. Muscle pain was lower initially but higher in second phase than skin.	PGE2 sensitizes proton-induced nociception, likely through TRPV1/ASIC-related peripheral nociceptor mechanisms relevant to inflammatory injection pain.
**30**	Sago et al. (2023) [51]	Referred sensation incidence, frequency, intensity, area; mechanical sensitivity	CPM response	Participants with referred sensations had increased mechanical sensitivity and decreased CPM (P<0.05). RS incidence, frequency, intensity, and area decreased during painful conditioning and after local anesthetic block (P<0.05).	Orofacial referred sensations depend on both peripheral afferent input and central descending inhibitory/convergence mechanisms.

**31**	Udagawa et al. (2018) [37]	eVAS pain intensity; NRS unpleasantness/tiredness/tension/soreness/stiffness; MPQ; pain drawings; pressure pain sensitivity	NA	Subcutaneous injections induced significantly more pain/unpleasantness than intramuscular injections; bone-surface glutamate increased pressure pain sensitivity more than intramuscular glutamate. Subcutaneous pain often described as sharp/pinching.	Chemical nociception and tissue depth jointly shape injection pain quality; subcutaneous and periosteal/bone-surface tissues may be more pain-sensitive than muscle in this model.
**32**	Uematsu et al. (2011) [52]	Pain ratings	fMRI BOLD activation	Nociceptive muscle stimuli activated anterior midcingulate, anterior/posterior insula, lentiform nucleus, thalamus, pre-SMA, DLPFC, and inferior parietal lobule. S2 was responsive to cutaneous pain rather than muscle pain.	Muscle and cutaneous pain differ in central processing patterns, indicating tissue-depth-specific pain networks.
**33**	Ushida et al. (2008) [18]	fMRI activation during virtual needle task	Emotional/cognitive activation inferred	Preconditioned group showed increased activation in anterior insula, inferior frontal gyrus, precentral gyrus, and cerebellum compared with non-preconditioned group; virtual needle task activated S2/premotor/parietal/temporal areas depending on group.	Needle-related pain can be amplified or represented centrally through visual threat, memory retrieval, and affective pain networks.
**34**	Verriotis et al. (2015) [17]	EEG event-related vertex potentials; Modified Behavioral Pain Scale (MBPS)	Behavioral pain score	Distinct inoculation event-related vertex potential observed after needle contact. Amplitudes of both negative-positive components were greater in 12-month infants; EEG amplitude and behavioral pain scores increased with age but were not correlated.	Real-life needle pain evokes measurable cortical nociceptive responses in infants; cortical processing changes with postnatal development.
**35**	Zijlstra et al. (2018) [38]	100-mm VAS pain sensation; acceptability	Injection-site reactions; backflow	Mean pain scores were generally <20 mm VAS. Pain increased with 2250 µL vs smaller volumes; 2250 µL increased pain vs 800 µL and needle-only; thigh injections more painful than abdomen by 2.1 mm (P=0.0013). Pain did not increase with speed; acceptance high (93.7–98.7%).	Large volume and thigh site increase SC injection pain, but absolute pain levels were low and speed had little clinical impact.

2D, two-dimensional; ASIC, acid-sensing ion channel; BOLD, blood-oxygen-level-dependent; C3a, complement component 3a; CCLAD, computer-controlled local anesthetic delivery; CI, confidence interval; CPM, conditioned pain modulation; CS, counter-stimulation; DLPFC, dorsolateral prefrontal cortex; EEG, electroencephalography; eVAS, electronic visual analog scale; FLACC, Face, Legs, Activity, Cry, Consolability scale; fMRI, functional magnetic resonance imaging; IM, intramuscular; LA, local anesthesia; LCT, long-chain triglyceride; LCT/MCT, long-chain triglyceride/medium-chain triglyceride; LS, least squares; MBPS, Modified Behavioral Pain Scale; MPQ, McGill Pain Questionnaire; NA, not applicable; NRS, numeric rating scale; P, probability value; PGE2, prostaglandin E2; pre-SMA, pre-supplementary motor area; RB, regular bevel; RS, referred sensation; S2, secondary somatosensory cortex; SB, scalpel bevel; SC, subcutaneous; SEM, Sound–Eye–Motor scale or scanning electron microscopy, depending on context; SpO2, peripheral oxygen saturation; TB, triple bevel; TRPV1, transient receptor potential vanilloid 1; VAS, visual analog scale; VCARS, Venham Clinical Anxiety Rating Scale; VR, virtual reality; WBFPS, Wong-Baker Faces Pain Scale.

Table S6. Risk-of-bias assessment matrix for the included studies.

Author/Year	Q1	Q2	Q3	Q4	Q5	Q6	Q7	Q8	Q9	Q10	Q11	Q12	Q13	% Yes	Overall risk
Alfaraj et al. (2022) [26]	-	+	-	-	-	-	-	+	-	x	?	?	?	20%	High
Ami et al. (2015) [28]	-	-	-	-	-	-	-	+	-	x	?	?	?	10%	High
Aminabadi et al. (2008) [40]	+	-	-	x	x	-	+	-	-	+	+	+	+	46%	High

Author/Year	Q1	Q2	Q3	Q4	Q5	Q6	Q7	Q8	Q9	Q10	Q11	Q12	Q13	% Yes	Overall risk
Aravena et al. (2018) [29]	+	-	+	x	x	-	+	+	+	+	+	+	+	69%	Moderate
Arendt-Nielsen et al. (2006) [9]	+	+	+	+	x	+	+	+	+	?	?	?	?	89%	Low
Badr et al. (2026) [41]	+	-	+	x	x	-	+	+	+	+	+	+	+	69%	Moderate
Barnhill et al. (1996) [42]	-	-	-	x	x	x	+	-	-	+	+	+	-	31%	High
Berteau et al. (2015) [30]	+	-	+	x	x	-	+	+	+	+	+	+	+	69%	Moderate
Dau et al. (2017) [16]	+	-	+	x	x	-	+	+	+	+	+	+	+	69%	Moderate
Flisfisch et al. (2021) [43]	-	-	+	x	x	-	+	+	-	+	+	+	+	54%	Moderate
Fowler-Kerry et al. (1987) [53]	+	-	+	x	x	-	+	+	-	+	+	+	+	62%	Moderate
Goodell et al. (2000) [44]	-	-	+	x	x	-	+	+	-	+	+	+	+	54%	Moderate
Hagwane et al. (2025) [45]	-	-	-	x	x	-	+	+	-	+	+	+	-	38%	High
Hambouta et al. (2025) [46]	+	-	+	x	x	-	+	+	+	+	+	+	+	69%	Moderate
Hegde et al. (2019) [47]	-	-	+	x	x	x	+	+	+	+	+	+	+	62%	Moderate
Heise et al. (2014) [12]	+	+	+	+	x	+	+	+	+	+	+	+	+	92%	Low
Jabeen et al. (2025) [48]	+	+	+	x	x	x	+	+	+	+	+	x	x	62%	Moderate

Author/Year	Q1	Q2	Q3	Q4	Q5	Q6	Q7	Q8	Q9	Q10	Q11	Q12	Q13	% Yes	Overall risk
Jin et al. (2025) [39]	-	+	-	-	-	-	+	+	-	x	?	?	?	30%	High
Karuppiah et al. (2026) [49]	+	+	x	x	x	+	+	+	+	+	+	+	+	77%	Moderate
Khodorova et al. (2010) [33]	-	+	-	-	-	-	-	+	-	-	?	?	?	20%	High
Kudo et al. (2005) [11]	+	-	+	+	x	+	+	+	+	?	?	?	?	78%	Moderate
Moaddabi et al. (2024) [34]	+	-	+	x	x	x	+	+	+	+	+	+	+	69%	Moderate
Nunna et al. (2019) [50]	+	-	x	x	x	-	+	+	+	+	+	+	+	62%	Moderate
Ohmizo et al. (2005) [35]	-	-	+	+	+	+	+	+	+	+	+	+	+	85%	Low
Padmanabh et al. (2025) [54]	+	-	+	x	x	x	+	+	+	+	+	+	+	69%	Moderate
Rukwied et al. (2007) [36]	+	-	+	+	x	-	+	+	+	+	+	+	+	77%	Moderate
Sago et al. (2023) [51]	+	+	+	+	+	+	+	+	+	?	?	?	?	100%	Low
Udagawa et al. (2018) [37]	+	-	+	+	x	+	+	+	+	+	+	+	+	85%	Low
Uematsu et al. (2011) [52]	+	+	+	+	x	+	+	+	x	?	?	?	?	78%	Moderate
Ushida et al. (2008) [18]	+	+	+	+	x	+	+	+	x	?	?	?	?	78%	Moderate
Verriotis et al. (2015) [17]	+	+	+	+	-	x	+	x	?	?	?	?	?	62%	Moderate

Author/Year	Q1	Q2	Q3	Q4	Q5	Q6	Q7	Q8	Q9	Q10	Q11	Q12	Q13	% Yes	Overall risk
Zijlstra et al. (2018) [38]	-	-	+	+	x	+	+	+	x	+	+	+	+	69%	Moderate

Table S7. Question key for the risk-of-bias assessment tools [22,23].

Checklist	Question	Assessment item
JBI RCT	Q1	Was true randomization used for assignment of participants to treatment groups?
	Q2	Was allocation to treatment groups concealed?
	Q3	Were treatment groups similar at baseline?
	Q4	Were participants blind to treatment assignment?
	Q5	Were those delivering treatment blind to treatment assignment?
	Q6	Were outcome assessors blind to treatment assignment?
	Q7	Were treatment groups treated identically other than the intervention of interest?
	Q8	Was follow-up complete and, if not, were differences between groups adequately described and analyzed?
	Q9	Were participants analyzed in the groups to which they were randomized?

Checklist	Question	Assessment item
	Q10	Were outcomes measured in the same way for treatment groups?
	Q11	Were outcomes measured reliably?
	Q12	Was appropriate statistical analysis used?
	Q13	Was the trial design appropriate and were deviations accounted for?
JBI Quasi-experimental	Q1	Is it clear what is the cause and what is the effect?
	Q2	Were the participants included in comparisons similar?
	Q3	Were participants included in comparisons receiving similar treatment/care, other than the exposure or intervention of interest?
	Q4	Was there a control group?
	Q5	Were there multiple measurements of the outcome both pre and post the intervention/exposure?
	Q6	Was follow-up complete and, if not, were differences between groups adequately described and analyzed?
	Q7	Were outcomes measured in the same way for participants included in comparisons?
	Q8	Were outcomes measured reliably?
	Q9	Was appropriate statistical analysis used?

Checklist	Question	Assessment item
JBI Analytical Cross-sectional	Q1	Were the criteria for inclusion in the sample clearly defined?
	Q2	Were the study subjects and the setting described in detail?
	Q3	Was the exposure measured in a valid and reliable way?
	Q4	Were objective, standard criteria used for measurement of the condition?
	Q5	Were confounding factors identified?
	Q6	Were strategies to deal with confounding factors stated?
	Q7	Were the outcomes measured in a valid and reliable way?
	Q8	Was appropriate statistical analysis used?
SYRCLE	Q1	Was the allocation sequence adequately generated and applied?
	Q2	Were the groups similar at baseline or were they adjusted for confounders?
	Q3	Was allocation adequately concealed?
	Q4	Were the animals randomly housed during the experiment?
	Q5	Were caregivers and investigators blinded to the intervention?
	Q6	Were animals selected at random for outcome assessment?

Checklist	Question	Assessment item
	Q7	Was the outcome assessor blinded?
	Q8	Were incomplete outcome data adequately addressed?
	Q9	Are reports free of selective outcome reporting?
	Q10	Was the study apparently free of other problems that could result in a high risk of bias?
